# hTERT-Immortalized Mesenchymal Stem Cell-Derived Extracellular Vesicles: Large-Scale Manufacturing, Cargo Profiling, and Functional Effects in Retinal Epithelial Cells

**DOI:** 10.3390/cells13100861

**Published:** 2024-05-17

**Authors:** Jessica Hindle, Anastasia Williams, Yuriy Kim, Dongsung Kim, Kajal Patil, Pooja Khatkar, Quinn Osgood, Collin Nelson, David A. Routenberg, Marissa Howard, Lance A. Liotta, Fatah Kashanchi, Heather Branscome

**Affiliations:** 1 ATCC, Manassas, VA 20110, USA; 2 Laboratory of Molecular Virology, School of Systems Biology, George Mason University, Manassas, VA 20110, USAkpatil3@gmu.edu (K.P.);; 3 Meso Scale Diagnostics, L.L.C., Rockville, MD 20850, USAdroutenberg@meso-scale.com (D.A.R.); 4 Center for Applied Proteomics and Molecular Medicine, George Mason University, Manassas, VA 20110, USA

**Keywords:** extracellular vesicles (EVs), hTERT-immortalized mesenchymal stem cells, retinal pigment epithelium (RPE) cells, ionizing radiation (IR)

## Abstract

As the economic burden associated with vision loss and ocular damage continues to rise, there is a need to explore novel treatment strategies. Extracellular vesicles (EVs) are enriched with various biological cargo, and there is abundant literature supporting the reparative and immunomodulatory properties of stem cell EVs across a broad range of pathologies. However, one area that requires further attention is the reparative effects of stem cell EVs in the context of ocular damage. Additionally, most of the literature focuses on EVs isolated from primary stem cells; the use of EVs isolated from human telomerase reverse transcriptase (hTERT)-immortalized stem cells has not been thoroughly examined. Using our large-scale EV-manufacturing platform, we reproducibly manufactured EVs from hTERT-immortalized mesenchymal stem cells (MSCs) and employed various methods to characterize and profile their associated cargo. We also utilized well-established cell-based assays to compare the effects of these EVs on both healthy and damaged retinal pigment epithelial cells. To the best of our knowledge, this is the first study to establish proof of concept for reproducible, large-scale manufacturing of hTERT-immortalized MSC EVs and to investigate their potential reparative properties against damaged retinal cells. The results from our studies confirm that hTERT-immortalized MSC EVs exert reparative effects in vitro that are similar to those observed in primary MSC EVs. Therefore, hTERT-immortalized MSCs may represent a more consistent and reproducible platform than primary MSCs for generating EVs with therapeutic potential.

## 1. Introduction

Mesenchymal stem cells (MSCs) are widely recognized for the various therapeutic applications they can offer to both science and medicine. Since their discovery in the late 1960s, MSCs have been extensively studied for their regenerative properties across various tissues; according to clinicaltrials.gov, over 1400 studies currently cross reference with “mesenchymal stem cells” [1,2]. Most recently, MSCs have also been proposed as an alternative therapy for the secondary complications associated with COVID-19 [3,4,5,6]. The therapeutic properties of MSCs have been attributed to their ability to influence and act upon cells within the adaptive and innate immune systems. Mechanistically, this is achieved through paracrine interactions where soluble factors mediate angiogenic, anti-apoptotic, anti-inflammatory, anti-oxidative, and other reparative effects in damaged or distressed cells [7,8,9]. Growing evidence now suggests that extracellular vesicles (EVs) are the secreted factors that aid in driving these effects. EVs represent a mixture of extracellular particles that display biochemical, biophysical, and functional heterogeneity and contribute to various homeostatic processes [10]. Historically, exosomes and microvesicles (MVs) comprised the two subtypes of stem cell EVs that have been the most widely studied for their physiological and pathological interactions [11,12]. However, in the 2024 Minimal Information for Studies of Extracellular Vesicles (MISEV) guidelines, researchers are discouraged from using these terms when describing EVs. Instead, it is recommended to cautiously use the operational terms “small EVs” (i.e., less than 200 nm in size) and “large EVs” (i.e., greater than 200 nm in size) when describing EV subtypes [13].

MSC EVs are enriched with various types of biological cargo, including nucleic acids, proteins, and lipids [14,15,16]. Upon fusion or uptake, EV-associated cargo is transferred to recipient cells where it has the potential to interact with numerous signal transduction pathways. For example, previous studies have demonstrated the involvement of MSC EVs in the mTOR, Akt, ERK, NF-kB, and MAPK signaling pathways [17,18,19,20,21,22]. Along these lines, there is abundant literature supporting the reparative (e.g., pro-survival, anti-apoptotic) and immunomodulatory properties of MSC EVs across a broad range of pathologies, including those affecting the central nervous system (CNS), liver, kidney, lung, and heart, as reviewed elsewhere [23,24]. While the literature surrounding MSC EV-mediated tissue repair is vast, one area that remains relatively underexplored is the therapeutic potential of stem cell EVs in the context of ocular damage or disease.

Recent reports have estimated that at least 1.1 billion individuals are affected by vision impairment and that the economic burden associated with vision loss is greater than USD 130 billion [25,26]. The Centers for Disease Control and Prevention list age-related diseases such as macular degeneration, cataracts, diabetic retinopathy, and glaucoma as the leading causes of impaired vision (CDC.gov). Additionally, combat ocular trauma represents a major cause of visual impairment and morbidity among members of the military [27]. Therefore, with a significant percentage of the population at risk for experiencing vision issues, there is an unmet need to explore novel treatment strategies.

Because of its classification as “immune privileged,” meaning that it is inherently protected from potential damage caused by inflammation, the eye is an attractive therapeutic target for stem cells and their byproducts (i.e., EVs). Along these lines, different types of stem cells, including neural stem cells, MSCs (bone marrow, adipose, dental pulp), and induced pluripotent stem cells (iPSCs), have been studied for their ability to repair retinal neurons, retinal pigment epithelial cells, and corneal endothelium. Additionally, a number of clinical trials using MSC-based treatment against various eye diseases such as glaucoma, retinitis pigmentosa, age-related macular degeneration, and diabetic retinopathy have been registered on clinicaltrials.gov [28,29].

Despite this progress, several challenges have limited the therapeutic outcomes of cell-based therapy for eye disease. For example, decreased self-renewal, cell death, and reactive gliosis have been reported post-transplantation. Additionally, other factors such as donor age and tissue source can dampen the functional effects of transplanted MSCs [30]. For these reasons, alternative approaches, such as MSC EVs, should be evaluated. To date, only a few studies have investigated and documented the reparative effects of MSC-derived EVs against eye damage and/or disease; most of these studies have been reviewed elsewhere [31,32]. 

The majority of the literature focuses on MSC EVs isolated from primary cultures; however, there are certain factors that must be considered when working with cells isolated directly from donor tissue. For example, there is inherent tissue variability when isolating stem cells from different donors/source material and this can further compound the downstream lot-to-lot variability in EV preparations. In addition, large-scale/continuous manufacturing is difficult because of the limited and finite cellular lifespan of primary stem cells. The “end replication problem” is the phenomenon by which telomeres are progressively shortened during DNA replication; over time, this leads to growth arrest and replicative senescence [33]. Previous research has demonstrated that human MSCs lack active expression of telomerase, the reverse transcriptase enzyme that is responsible for maintaining telomere length [34,35,36].

To overcome these challenges, researchers have developed technology that allows for the continuous replication of primary cells. This technology, known as human telomerase reverse transcriptase (hTERT) immortalization, provided a breakthrough in primary cell research. Through the forced overexpression of exogenous hTERT, it was shown that telomere length could be preserved and cellular senescence could be prevented [37,38]. Since its inception, hTERT immortalization has been successful in immortalizing a wide range of human primary cells, including MSCs, thereby extending their proliferative abilities in vitro [39,40,41,42,43]. However, it is worth noting that there have been conflicting results regarding whether or not hTERT immortalization may predispose cultures to a cancerous or malignant phenotype, as some of the literature has suggested that hTERT immortalization can contribute to genomic instability and promote premalignant phenotypes and cancer invasion [44,45,46]. Furthermore, whether or not the EVs isolated from these cell types exhibit similar effects remains to be explored.

In this study, we sought to characterize EVs from hTERT-immortalized MSCs and to evaluate their reparative effects on retinal epithelial cells in vitro. As a control, we performed side-by-side studies with EVs from cancer cells to compare their biochemical and functional properties. Using our large-scale EV manufacturing platform, we manufactured two independent batches (i.e., biological replicates) each of EVs from hTERT-immortalized MSCs and PC3 cancer cells. We employed various methods and technologies to characterize and profile EV-associated cargo. We also utilized several cell-based assays to compare the effects of these EVs on both healthy and damaged retinal pigment epithelial cells. The results from our studies not only highlight the differences in EV-associated cargo and functional effects but also suggest that hTERT-immortalized MSCs may represent a more consistent and reproducible platform than primary MSCs for generating EVs with therapeutic potential.

## 2. Materials and Methods

### 2.1. Cell Lines

The following cell lines from the American Type Culture Collection (ATCC, Manassas, VA, USA) were used for downstream EV isolation: hTERT-immortalized MSCs (ASC52telo; ATCC^®^ SCRC-4000™), prostate carcinoma cells (PC3; ATCC^®^ CRL-1435™), and colorectal carcinoma cells (HCT 116; ATCC^®^ CCL-247™). hTERT-immortalized MSCs were cultured in Mesenchymal Stem Cell Basal Medium (ATCC^®^ PCS-500-030™) supplemented with the Mesenchymal Stem Cell Growth Kit (ATCC^®^ PCS-500-040™) and G418 at a final concentration of 0.2 mg/mL. PC3 cells were cultured in F-12K Medium (ATCC^®^ 30-2004™) supplemented with 10% Fetal Bovine Serum (FBS) (ATCC^®^ 30-2002™). HCT 116 cells were cultured in McCoy’s 5A Medium (ATCC^®^ 30-2007™) supplemented with 10% FBS. The human retinal pigmented epithelium cell line ARPE-19 (ATCC^®^ CRL-2302™) was cultured in DMEM:F12 Medium (ATCC^®^ 30-2006™) supplemented with 10% FBS. All cell lines were maintained in culture following the manufacturer’s recommended guidelines.

### 2.2. EV Isolation and Nanoparticle Tracking Analysis

Cells were maintained at 37 °C and 5% CO_2_ and were scaled up according to the manufacturer’s recommended guidelines in T-flasks and Corning^®^ CellSTACK^®^ (Corning, NY, USA) vessels until an approximate volume of five liters was achieved. Two batches of EVs (i.e., Lot 1 and Lot 2) were independently manufactured from each cell type and therefore represented biological replicates. Prior to EV isolation, cells were cultured in EV-depleted FBS. EV isolation was based on our previously published protocols [47]. Briefly, this process involves centrifugation (i.e., 2000× *g*) of the bulk cell culture medium followed by tangential flow filtration (TFF) (KrosFlo^®^ Research Iii, Repligen Corporation, Waltham, MA, USA) using a modified polyethersulfone (mPES) membrane with a molecular weight cutoff of 500 kDa. The samples underwent a 5X buffer exchange/diafiltration with PBS prior to the final concentration and then were sterile-filtered (i.e., 0.2 µM) to obtain the concentrated product [47]. The EVs used in this study are available from ATCC as follows: hTERT -immortalized MSC (ATCC^®^ SCRC-4000-EXM™), PC3 (ATCC^®^ CRL-1435-EXM™), and HCT 116 (ATCC^®^ CCL-247™). EV aliquots were frozen at −20 °C prior to downstream applications. Nanoparticle tracking analysis (NTA) was performed using a NanoSight^®^ NS300 (Malvern Panalytical, Malvern, Worcestershire, UK) to measure EV concentration (particles/mL) and size distribution. This equipment is routinely maintained in accordance with the manufacturer’s recommendations. Two biological replicates (i.e., Lot 1 and Lot 2) of each EV sample were analyzed. EVs were diluted in sterile PBS prior to analysis, and each EV sample was measured in triplicate. The data were analyzed using the instrument’s built-in NanoSight^®^ NTA software version 3.4.

### 2.3. Transmission Electron Microscopy

Approximately five mL of each EV suspension was applied for five minutes onto formvar/carbon-coated 200 mesh copper grids (Electron Microscopy Sciences) that were glow-discharged (20 mA for 60 s) right before use. EVs were negatively stained with 1% uranyl acetate (Electron Microscopy Sciences, Hatfield, PA, USA) for one minute and air-dried before imaging. A FEI Talos™ F200X transmission electron microscope operated at 200 kV was used. Images were acquired with a Thermo Scientific™ Ceta 16M CMOS (Waltham, MA, USA) camera at a relative magnification of 58,000× (field of view 883.1 nm, pixel size 215.6 pm, defocus ~1 mm). Thermo Scientific Maps Software version 3.18 was used for the automated acquisition of 20 × 20 tiled images to create a large field of view. Representative images were trimmed from large tiles using Adobe Photoshop, modifying brightness and contrast if necessary.

### 2.4. Multiplex Immunoassays

Total levels of intact EVs in each sample were compared using R-PLEX^®^ EV assays (Meso Scale Discovery^®^, Rockville, MD, USA) in a multiplex format according to the manufacturer’s instructions. Capture antibodies for CD63, CD81, and CD9 were displayed on MULTI-SPOT^®^ U-PLEX^®^ plates (Meso Scale Discovery^®^, Rockville, MD, USA), and EVs were captured and subsequently detected with a cocktail of ECL-labeled CD63, CD81, and CD9 antibodies. The ECL signal produced on each spot was proportional to the relative number of EVs in the sample presenting the target of each capture antibody. An EV calibrator provided by the manufacturer was also assayed, but since the signals for all samples were in the linear range of each assay, we reported raw ECL counts rather than relative concentrations. All assays were performed in duplicate for each lot.

EVs were screened for thirty additional surface markers using a similar multiplex immunoassay. These markers were AAP, ALCAM, ALK7, α-sarcoglycan, CD15, CD24, CD276, CD40, CEA, DPPIV, EGFR, CD105, EpCAM, EPCR, EphA2, ESAM, FAP-α, FASR, GJA1, HCAM, ICAM1, ITGB1, MCAM, Neprilysin, CD73, PECAM, Thrombomodulin, Thy-1, and TNAP. Here, capture antibodies for up to 9 putative EV surface antigens and a negative control capture antibody were displayed on MULTI-SPOT^®^ U-PLEX^®^ (Meso Scale Discovery^®^, Rockville, MD, USA) plates. EVs from each sample were captured and subsequently detected with a cocktail of ECL-labeled CD63, CD81, and CD9 antibodies. The ECL signal produced on each spot was proportional to the relative number of EVs in the sample presenting the target of each capture antibody. Assays were performed in duplicate for each lot.

Inflammatory cytokines were assayed in the EV samples using the S-PLEX^®^ proinflammatory Panel 1 Kit (Meso Scale Discovery^®^, Rockville, MD, USA) following the manufacturer’s instructions. Assays were performed in duplicate for each lot. Where detectable, analyte concentrations were calculated using a 4-PL fit of a standard curve of the calibrators. EVs were assayed before and after lysis using 0.5% Triton X-100 to determine whether cytokines were potentially encapsulated within the EV membrane.

For all multiplex immunoassays, EV input was based on volume (approximately 25 µL per sample).

### 2.5. Western Blot

Protein concentration was measured by the Bicinchoninic acid (BCA) assay. EV lysates (approximately 3 µg per sample) and cell lysates (approximately 10 µg per sample) were mixed with Laemmli buffer and heated at 95 °C. Samples were run on a 4–20% Tris/glycine gel (Invitrogen, Carlsbad, CA, USA) and then transferred to a PVDF membrane overnight. Blocked membranes were incubated with the appropriate primary antibody overnight at 4 °C. The primary antibodies used included α-CD63, α-CD81, α-CD9, α-GAPDH, α-Caspase 3, α-Cyclin A, α-Cyclin D1, α-Cyclin E, α-N-cadherin, α-E-cadherin, α-CD142, α-VEGF, α-DNA Polymerase β, α-p-AKT, and α-Akt, and α-Actin. See Table 1 below for primary antibody catalog information. After incubation with the appropriate secondary antibody, membranes were developed with Clarity™ or Clarity Max™ Substrate (Bio-Rad, Hercules, CA, USA), and images were taken with the ChemiDoc™ Imaging System (Bio-Rad). Images of full-length blots are included in Figure S1.

### 2.6. Proteomics

Purified EV samples were processed and analyzed by mass spectrometry according to the methods described in Howard et al. [48]. Liquid chromatography coupled tandem mass spectrometry (LC-MS/MS) experiments were performed on an Exploris™ 480 (Thermo Fisher Scientific^®^, Waltham, MA, USA) operated in a data-dependent mode. Tandem mass spectra were searched against the NCBI human database using Proteome Discover v 2.3 from Thermo Fisher Scientific^®^. A 1% false discovery rate (FDR) was used as a cutoff value for reporting peptide spectrum matches (PSM) from the database search. Shared proteins between EVs were analyzed using the web-based Venn diagram maker InteractiVenn (https://www.interactivenn.net) [49]. The proteomics data files were deposited to the ProteomeXchange Consortium via the PRIDE partner repository with the dataset identifier PXD044629.

The Search Tool for the Retrieval of Interacting Genes (STRING) was used to calculate the protein–protein interactions (PPIs) of peptides extracted from the EV preparations. STRING is a publicly accessible database of known and projected PPIs. Direct (physical) and indirect (functional) linkages result through computer predictions, information transmission between species, and interactions collated from primary databases (i.e., Biocarta, BioCyc, KEGG, and Reactome). PDB, BioGRID, IntAct, and other databases provide experimental and biochemical data. In this study, accession numbers of unique peptides associated with hTERT-immortalized MSC EVs and PC3 EVs were imported into STRING; interactions with a combined score of 0.70 (high confidence) were considered significant.

### 2.7. RNA Extraction and qPCR

RNA was isolated from cell pellets and EVs using TRIzol (Invitrogen, Carlsbad, CA, USA) according to the manufacturer’s protocol. The RNA samples were enriched for mRNA using Oligo (dT) 25 magnetic beads (Catalog S1419S, New England Biolabs, Ipswich, MA, USA) according to the manufacturer’s guidelines. Briefly, isolated RNA samples were incubated with Oligo (dT) beads, washed, and eluted in Tris-HCl. GoScript™ Reverse Transcriptase (Promega, Madison, WI, USA) was used for cDNA synthesis along with oligo (dT) reverse primers (Promega). For qPCR, cDNA samples were prepared with an SYBR^®^ Green (Bio-Rad) master mix with the corresponding primer sets. The primers and annealing temperatures (Tm) are shown in Table 2. The reaction conditions included the following: 50 °C for 2 min (1 cycle), 95 °C for 2.5 min (1 cycle), 95 °C for 15 s, and the corresponding annealing temperature for 40 s (41 cycles). All reactions were performed in triplicate. The data were quantified by comparison of cycle threshold (Ct) values generated by the BioRad CFX Manager^TM^ Software version 3.0. Sample input for cDNA synthesis and qPCR were standardized based on volume. The data were analyzed in GraphPad Prism.

### 2.8. Cell Migration Assay

ARPE-19 cells were seeded at an approximate density of 1.0 × 10^5^ viable cells/cm^2^ into cell culture inserts in their complete culture medium. Cells were incubated overnight and, upon reaching confluence, an artificial gap approximately 500 µm in diameter was introduced to the cultures by removing the inserts. Cells were gently rinsed once with PBS and then basal DMEM:F12 was added to each well. Untreated cells received DMEM:F12 only and EV-treated cells received either hTERT-immortalized MSC EVs or PC3 EVs at an approximate ratio of 1:10,000 (recipient cell:EV). Each condition was performed in duplicate, and cells were further incubated for a period of 48 h. Cells were imaged using the BioTek^®^ Cytation™ Cell Imaging Multimode Reader (Agilent, Santa Clara, CA, USA) at different time points. The percentage of gap coverage was quantified using ImageJ software version 1.54d and the phantast plugin [50].

### 2.9. In Vivo Wound Healing Assay

The Institutional Animal Care and Use Committee at ATCC approved all protocols. BALB/c mice (6 to 8 weeks old) were allowed to acclimate for a period of one week prior to initiating the study. The dorsal skin of each mouse was shaved twenty-four hours prior to the punch biopsy. On day 0, mice were anesthetized with Ketamine, and the surgical site was scrubbed with chlorhexidine scrub and alcohol. Two superficial wounds on the upper dorsal region were created using a 6 mm Disposable Biopsy Punch (Integra™ Miltex^®^, Princeton, NJ, USA). Each wound bed was immediately treated with four subcutaneous injections of the indicated treatments as follows: untreated (PBS), hTERT-immortalized MSC low dose (10 µg), hTERT-immortalized MSC high dose (100 µg), PC3 low dose (10 µg), and PC3 high dose (100 µg). EV treatments were based on approximate protein concentrations. All treatments were administered in a total volume of 200 µL with *n* = 5 mice per group. On day 2, all treatments were readministered as described above. The approximate diameter of each wound bed was measured immediately after administration (day 0) with a caliper, and subsequent measurements were taken and recorded daily.

### 2.10. Ionizing Radiation (IR)

ARPE-19 cells were seeded in either 96-well plates or 6-well plates. Semi-confluent cultures (i.e., ~60–70% confluent) were irradiated at a dose of 20 Gy using the RS 2000 X-ray Irradiator (Rad Source Technologies, Buford, GA, USA). Immediately after exposure, ARPE-19 cells were treated with either hTERT-immortalized MSC EVs or PC3 EVs (day 0) at an approximate ratio of 1:10,000 (recipient cell:EV). Untreated cells received culture medium only. Each condition was performed in duplicate, and cells were incubated for downstream assays.

### 2.11. Cell Viability and Repair Assays

ARPE-19 cells were seeded and irradiated as described above and incubated for either 5 days or 7 days. Cultures that were incubated for 5 days post-IR received only 1 dose of the indicated treatment on day 0. On day 5, cells that were seeded in 96-well plates were assayed for viability using the CellTiter-Glo^®^ Luminescent Cell Viability Assay (Promega, Madison, WI, USA) GloMax^®^-Multi Detection System (Promega). On day 5, cells that were seeded in 6-well plates were harvested, and protein lysates were prepared for Western blot. Cultures that were incubated in 6-well plates for 7 days post-IR received EVs on day 0 as well as additional EV treatments (same ratio) on days 1, 2, 3, and 6. On day 7, cells were imaged with the BioTek^®^ Cytation™ Cell Imaging Multimode Reader (Agilent, Santa Clara, CA), and then cells were harvested and protein lysates were prepared for Western blot.

For the inactivation of EV-associated proteins, EV samples were heated at 56 °C for approximately 30 min. For the inactivation of EV-associated RNAs, EV samples were treated with 45 MJ/cm^3^ of Ultraviolet-C (UV-C) light.

### 2.12. Statistical Analysis

Quantitative data were analyzed with GraphPad Prism software version 9.0. *p*-values were determined by either an unpaired *t*-test, one-way analysis of variance (ANOVA), or two-way ANOVA. *p*-values were defined as statistically significant (<0.05), of greater significance (<0.01), of greatest significance (<0.001), and of extreme significance (<0.0001).

## 3. Results

### 3.1. Large-Scale EV Isolation and Characterization

The ability to manufacture EVs at scale in a reproducible and robust manner remains a challenge for many EV researchers. We have previously published data demonstrating our ability to isolate EVs from large batches (i.e., multiple liters) of conditioned culture medium from A549 lung carcinoma cells, primary MSCs, and human induced pluripotent stem cells [47]. To further demonstrate our large-scale EV manufacturing capabilities, we isolated EVs from bulk suspensions of hTERT-immortalized MSCs and PC3 cancer cells. EVs were isolated as previously described with slight modifications to the protocol to further enhance the concentration of the final product [47].

**Figure 1:**
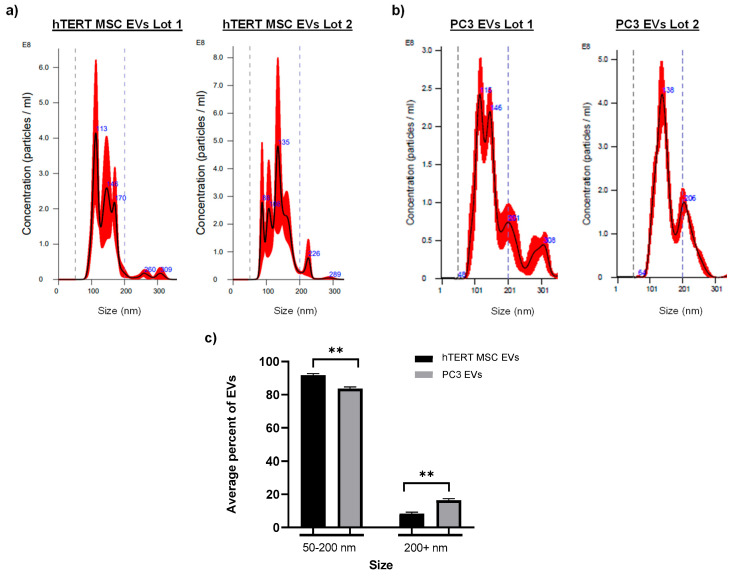

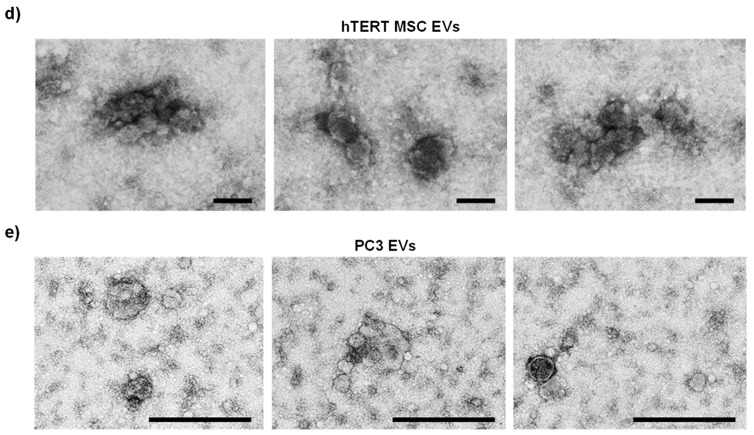
Nanoparticle tracking analysis and EV morphology. NTA was performed on two biological replicates (i.e., Lot 1 and Lot 2) of (**a**) hTERT-immortalized MSC EVs and (**b**) PC3 EVs to analyze relative concentration (particles/mL) and size distribution. Dashed lines indicate values of 50 nm and 200 nm. (**c**) The average % of EVs from each lot that measured between 50–200 nm in size and 200+ nm in size. *n* = 6 (2 biological replicates and each biological replicate was measured in triplicate). ** = *p* < 0.01. Representative TEM images show the appearance of (**d**) hTERT-immortalized MSC EVs and (**e**) PC 3 EVs. Scale bar = 100 nm.

To highlight the reproducible nature of our protocols, we included data from two independently produced large-scale batches of EVs isolated from each cell type. EV concentration and size distribution were analyzed by NTA. Dashed lines at 50 nm and 200 nm are included on each histogram as a visual tool to annotate the size distribution range that is expected to represent a population of small EVs. Figure 1a shows the NTA histograms for two lots of hTERT-immortalized MSC EVs, which display major peaks at 113 nm and 135 nm as well as several other prominent peaks less than 200 nm. Figure 1b shows the NTA histograms for two lots of PC3 EVs, which display major peaks at 115 nm and 138 nm. Both lots of PC3 EVs also displayed peaks of larger vesicles or aggregates greater than 200 nm. When comparing the average percentage of EVs among each lot within a defined size range (i.e., 50 to 200 nm and 200+ nm), the differences between hTERT-immortalized MSC EVs and PC3 EVs were significantly different. hTERT-immortalized MSC EVs displayed a higher percentage of EVs within the range of 50 to 200 nm, whereas PC3 EVs displayed a higher percentage of EVs larger than 200 nm (Figure 1c). These results are further supported by data in Figure S2a,b, which include the NTA profiles and size distribution measurements, respectively, of two independent batches of EVs isolated from the cancer cell line HCT 116. Similar to PC3 EVs, HCT 116 EVs show major peaks around 158 nm and 113 nm as well as other prominent peaks of larger vesicles greater than 200 nm. Again, when comparing the average percentage of EVs among each lot within defined size ranges, the differences between hTERT-immortalized MSC EVs and HCT 116 EVs were significantly different. hTERT-immortalized MSC EVs displayed a higher percentage of EVs within the range of 50 to 200 nm, whereas HCT 116 EVs displayed a higher percentage of EVs larger than 200 nm.

Transmission electron microscopy (TEM) was also performed on hTERT-immortalized MSC EVs and PC3 EVs to assess morphology. Representative images of hTERT-immortalized MSC EVs (Figure 1d) and PC3 EVs (Figure 1e) show the presence of vesicles that appear to be approximately 100 nm or smaller in diameter. Overall, hTERT-immortalized MSC EVs appeared to cluster in aggregates of small EVs with similar morphology, whereas PC3 EVs displayed more complex morphology and inconsistent structures.

**Figure 2:**
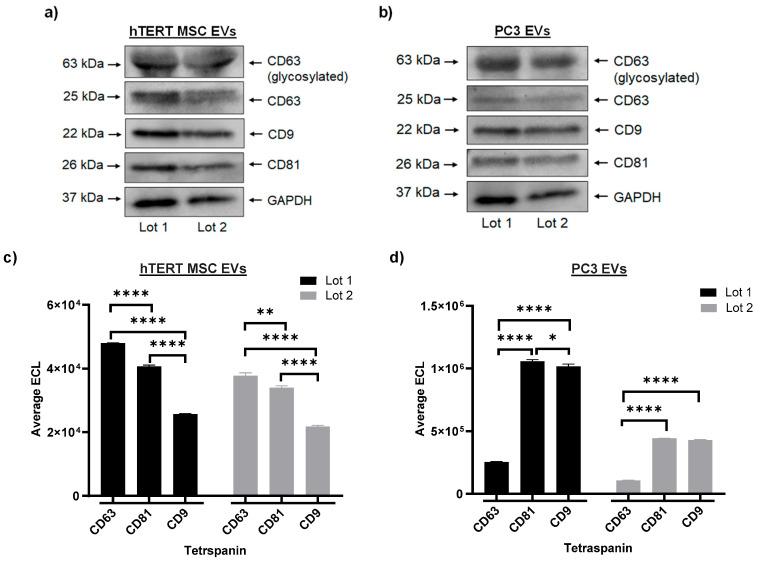
Characterization of EVs. (**a**) Western blot for CD63, CD9, CD81, and GAPDH from two independent lots (biological replicates) of hTERT-immortalized MSC EVs. (**b**) Western blot for CD63, CD9, CD81, and GAPDH from two independent lots (biological replicates) of PC3 EVs. Multiplex analysis was performed to analyze tetraspanins (CD63, CD81, and CD9). (**c**) Average ECL values from two independent lots (biological replicates) of hTERT-immortalized MSC EVs. Each lot was assayed in duplicate. (**d**) Average ECL values from two independent lots (biological replicates) of PC3 EVs. Each lot was assayed in duplicate. (* *p* < 0.05; ** *p* < 0.01; **** *p* < 0.0001).

EV protein concentration was measured by BCA. The average protein concentration of hTERT MSC EVs was 5.4 µg/µL (SD = 0.314), and the average protein concentration of PC3 EVs was 0.41 µg/µL (SD = 0.008). A Western blot was performed to assess the expression of tetraspanins between two independent lots of hTERT-immortalized MSC EVs and PC3 EVs. The results from this experiment show that both lots of hTERT-immortalized MSC EVs (Figure 2a) and both lots of PC3 EVs (Figure 2b) co-express CD63, CD9, and CD81, as well as GAPDH. To further assess the relative expression of EV-associated tetraspanins, EVs were assayed using a highly sensitive multiplex assay system. As shown in Figure 2c, both lots of hTERT-immortalized MSC EVs displayed consistent patterns of expression, with CD63 displaying the highest ECL values followed by CD81 and CD9 (CD63 > CD81 > CD9). The differences in ECL values were statistically significant between both lots. In contrast, Figure 2d shows that both lots of PC3 EVs displayed the opposite pattern of expression, with CD81 having the highest ECL values followed by CD9 and CD63 (CD81 > CD9 > CD63). Additionally, the average ECL of CD63 was significantly lower than both CD81 and CD9 in each lot of PC3 EVs. A similar pattern of expression was also observed in the two independent lots of HCT 116 (cancer) EVs (Figure S2c). The authors acknowledge that this trend in tetraspanin expression is based on an input of standardized volume, and further confirmations can be performed by standardizing sample input on other parameters such as EV number or EV protein.

Collectively, these results demonstrate our ability to consistently manufacture EVs from multiple different cell types with low lot-to-lot variability. These results also indicate that EVs from cancer cell lines contain subpopulations of larger vesicles (>200 nm) as compared with EVs from hTERT-immortalized MSCs. Lastly, these results suggest that EVs from cancer cells and stem cells display cell type-specific expression of CD63, CD9, and CD81. Given the reproducible nature of these data, all subsequent experiments will include data representing one lot of hTERT-immortalized MSC and PC3 EVs.

### 3.2. Profiling of EV Surface Proteins and EV-Mediated Effects on Cell Migration

The EV surface can be characterized by several different molecular components including receptors, transporters, enzymes, and antigens. In addition to aiding in EV identification, classification, and isolation, these molecules also play important biological roles by contributing to processes related to intercellular interactions and biodistribution [51]. To further explore the potential cargo differences between hTERT-immortalized MSC and cancer EVs, we performed EV surface marker screening using the U-PLEX^®^ multiplex assay platform. For these experiments, EVs were screened using antibodies targeting various cellular surface proteins and adhesion molecules expected to be present on EVs from some samples but not others. Our rationale for these studies was based on the premise that EV surface proteins may not only serve as biomarkers for disease but also be responsible for important functions relating to EV trafficking and uptake by recipient cells [52]. EVs presenting 24 of the 30 surface markers were present in one or both samples while α-Sarcoglycan, EPCR, ESAM, GJA1, PECAM, and TNAP were absent from both sample types.

**Figure 3:**
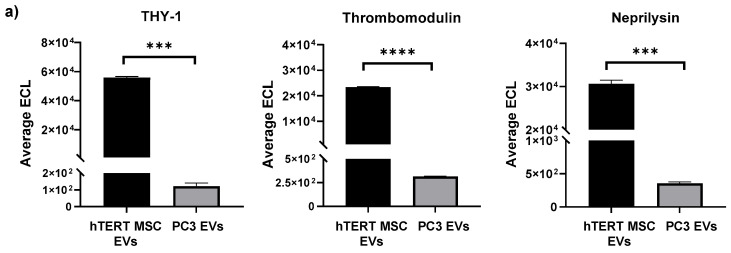

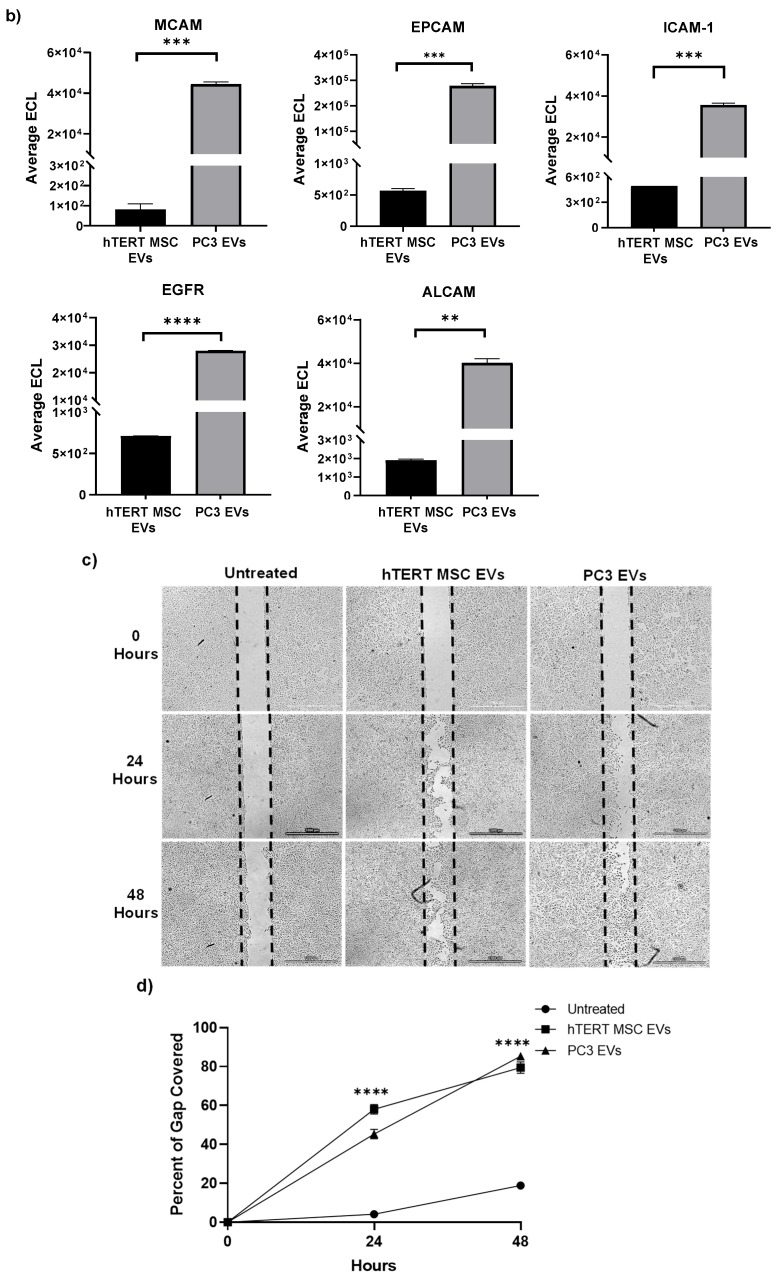
EV surface marker profiling and cell migration Assay. Multiplex analysis was performed to analyze various EV-associated surface marker proteins. Assays were run in duplicate. The average ECL values of each surface marker were analyzed and compared between hTERT-immortalized MSC and PC3 EVs. (**a**) THY-1, Thrombomodulin, and Neprilysin were significantly enriched in hTERT-immortalized MSC EVs. (**b**) MCAM, EPCAM, ICAM-1, EGFR, and ALCAM were significantly enriched in PC3 EVs. **** *p* < 0.0001; *** *p* < 0.001; ** *p* < 0.01. (**c**) A cell migration assay was performed using RPE cells. After the creation of an artificial gap, cells were immediately treated with EVs at an approximate ratio of 1:10,000 (recipient cell:EV). Representative images display gap closure over 48 h in response EVs. Scale bar = 1000 µm. (**d**) GraphPad Prism was used to analyze raw images to quantify percent gap coverage. **** *p* < 0.0001 relative to untreated.

When plotting the average ECL values of the screened proteins, we observed distinct differences between hTERT-immortalized MSC EVs and PC3 EVs. For example, the cell surface glycoprotein THY-1 (CD90) and the transmembrane proteins Neprilysin and Thrombomodulin (CD141) were strongly associated with hTERT-immortalized MSC EVs and displayed approximate fold-differences of 461, 85, and 72, respectively, when compared with those of PC3 EVs (Figure 3a). On the other hand, PC3 EVs were found to be significantly enriched with the transmembrane proteins MCAM (CD146), EpCAM (CD326), ICAM-1 (CD54), EGFR, and ALCAM (CD166) and displayed approximate fold-differences of 549, 489, 70, 39, and 21, respectively, when compared with those of hTERT-immortalized MSC EVs (Figure 3b). Comparison of the average ECL values between hTERT-immortalized MSC EVs and HCT116 EVs yielded similar results. Here, the values associated with THY-1, Thrombomodulin, and Neprilysin were each significantly higher in hTERT-immortalized MSC EVs (Figure S3a), whereas the values associated with MCAM, EPCAM, ICAM-1, ALCAM, and EGFR were all significantly higher in HCT116 EVs (Figure S3b). Not surprisingly, each of the surface proteins that are highly enriched in PC3 and HCT116 EVs are well-known markers that are associated with cancer and metastasis [53,54,55,56,57,58,59,60]. Interestingly, THY-1, Thrombomodulin, and Neprilysin, which were more highly enriched in hTERT-immortalized MSC EVs, may have potential roles in biological processes related to inflammation, tumor suppression, cellular differentiation and migration, regeneration, and apoptosis [61,62,63,64,65,66,67]. The authors acknowledge that the observed expression of EV-associated surface markers is based on an input of standardized volume, and further confirmations can be performed by standardizing sample input on other parameters such as EV number or EV protein.

Given the data suggesting that both hTERT-immortalized MSC and PC3 EVs contain proteins and molecules that promote various cellular processes, we next evaluated their effects on the migration of retinal pigment epithelial (RPE) cells in vitro. For this assay, an artificial gap was introduced to confluent cultures of RPEs and immediately treated with EVs. Cells were further incubated and observed for a period of two days, and the representative images in Figure 3c show a decrease in the gap width of cells that were treated with both EVs relative to the untreated cells. We next used ImageJ to quantify the percent of gap coverage. Image analysis revealed that after 48 h, the percent of gap coverage was approximately 18%, 79%, and 85% for the untreated, hTERT-immortalized MSC EV, and PC3 EV treated cells, respectively. At each time point (24, 48 h), treatment with both EV types resulted in a significant increase in cell migration relative to untreated cells. At 24 h post-treatment, there was a significant difference between the effects of hTERT-immortalized MSC EVs (58% gap coverage) and PC3 EVs (45% gap coverage); however, there was no statistically significant difference between cells treated with hTERT-immortalized MSC EVs and PC3 EVs after 48 h (Figure 3d). These data confirm that both hTERT-immortalized MSC EVs and PC3 EVs can promote the migration of RPE cells in vitro.

The effects of EVs on cell migration were further confirmed in vivo using a mouse model for wound healing. In this study, two dorsal punch biopsies were introduced per mouse (day 0) and each wound bed was immediately treated with four subcutaneous injections of EVs (Figure S4a). EV treatments were administered at both low (10 µg total per wound bed) and high (100 µg total per wound bed) doses for both hTERT-immortalized MSC and PC3 EVs. Untreated control mice received four subcutaneous injections of PBS around each wound bed. All treatments were readministered as described above on day 2. The diameter of each wound bed was measured over a period of 9 days. By day 6, the average wound diameters of mice treated with both doses of hTERT-immortalized MCS EVs were significantly less than those of untreated mice. On the other hand, only the low dose of PC3 EVs resulted in a significant reduction in the wound diameter by day 6 (Figure S4b). By day 9, only the high dose of hTERT-immortalized MSC EVs had a significant effect on the reduction in the average wound diameter relative to untreated. We also observed that the average wound diameters of the PC3 EV high dose and untreated control were relatively similar on day 9 (Figure S3c). Taken together, these data point toward a time- and dose-effect response of hTERT-immortalized MSC EVs on wound healing in vivo; furthermore, these data suggest that the effects of cancer EVs on cell migration may be more variable than the effects of hTERT-immortalized MSC EVs.

### 3.3. Effects of hTERT-Immortalized MSC EVs on Cell Viability and Apoptosis in RPE Cells after Ionizing Radiation

Based on the above data demonstrating that hTERT-immortalized MSC EVs can promote processes related to cell migration, we next sought to investigate the reparative properties of these EVs on damaged RPE cells in vitro. Ionizing radiation (IR) is well known for inducing a cascade of biological effects in cells and tissues including DNA damage, chromosomal abnormalities, inflammation, apoptosis, and carcinogenesis [68,69]. In addition, our previous research suggested that stem cell EVs can rescue the viability of multiple cell types (neurons, astrocytes, and monocyte-derived macrophages) after exposure to ionizing radiation (IR) [15]. Therefore, we chose to adopt a similar model of IR-induced damage using RPE cells for these experiments.

**Figure 4:**
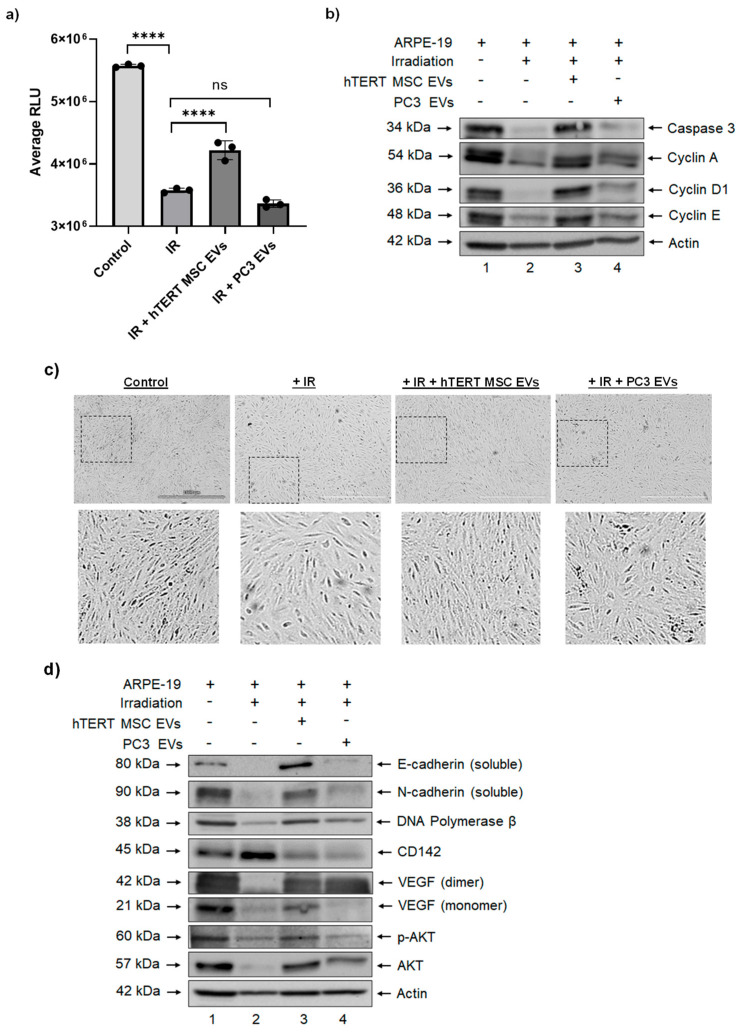
Functional effects of EVs on irradiated RPEs. RPE cells were exposed to IR and treated with EVs (day 0). (**a**) On day 5, a CellTiter-Glo^®^ assay was performed to quantify the effects of EVs on cellular viability. *n* = 3. **** *p* < 0.0001. (**b**) A Western blot was performed to evaluate the effects of EVs on the expression of proteins involved in apoptosis and cell cycle regulation. (**c**) RPEs received additional EV treatments on days 1, 2, 3, and 6. Representative photographs show their relative appearance on day 7. Scale bar = 1000 µm. (**d**) Day 7 cultures were harvested, and a Western blot was performed to evaluate the effects of EVs on the expression of various proteins involved in cellular migration and repair.

We first carried out a titration study with RPE cells to determine an acceptable dose of IR and from these studies, we chose a dose of 20 Gy for downstream experiments. To evaluate the effects of EVs on irradiated cells, semi-confluent (i.e., 60–70%) cultures of RPE cells were exposed to 20 Gy IR and immediately treated with EVs (Day 0). Cell viability was assayed on day 5 post-irradiation. The results from this experiment confirmed that exposure of RPE cells to IR resulted in a significant reduction in viability relative to control cells and that a single treatment with hTERT-immortalized MSC EVs had a significant impact on restoring cellular viability (Figure 4a). Interestingly, the viability of irradiated cells that received a single treatment with PC3 EVs was lower than the viability of the irradiated cells. 

To better assess the effects of EVs on the health and growth of RPE cells, we performed a Western blot on protein lysates from day 5 cultures. As shown in Figure 4b, exposure of RPE cells to irradiation was associated with decreased expression of Caspase 3 (uncleaved). The addition of hTERT-immortalized MSC EVs to irradiated RPE cells restored the expression of this protein to a level that was comparable to the non-irradiated cells, suggesting an anti-apoptotic effect. However, the addition of PC3 EVs had little to no effect on Caspase 3 expression relative to the irradiated cells. 

We were also interested in evaluating the potential cell cycle dynamics of EV-treated cells post-irradiation. To this end, we assessed the relative expression of proteins that are responsible for regulating progression through the cell cycle. The results in Figure 4b show that exposure of RPE cells to irradiation led to decreased expression of Cyclin A, Cyclin D1, and Cyclin E relative to non-irradiated cells. Again, the addition of hTERT-immortalized MSC EVs to irradiated cells was associated with a reversal of this effect as indicated by increased expression of Cyclin A, Cyclin D1, and Cyclin E relative to irradiated cells. Although PC3 EVs were also associated with slight increases in Cyclin A, Cyclin D1, and Cyclin E, their relative expression was still lower when compared with hTERT-immortalized MSC-EV treated cells. These data suggest that EVs from stem cells and cancer cells may exert differential effects on the regulation of the cell cycle in damaged cells.

### 3.4. hTERT-Immortalized MSC EVs Rescue the Expression of Cell Migration and Repair Proteins in RPE Cells after Ionizing Radiation

Our studies so far focused on the effects of a single treatment of EVs and, remarkably, the data suggest that a single dose is sufficient to exert pro-migratory and anti-apoptotic effects in retinal epithelial cells. We next asked whether multiple consecutive EV treatments would still promote a reparative phenotype in IR-damaged RPEs and if we would observe any adverse effects that would be indicative of a potential loss of cellular homeostasis. For this set of experiments, RPEs were exposed to IR (20 Gy) and immediately treated with EVs (day 0). Cells received additional EV treatments on days 1, 2, 3, and 6 for a total of five treatments post-IR. 

On day 7, cultures were observed and representative photographs were taken. As shown in Figure 4c, when the images were enlarged, the control (non-irradiated) cells exhibited a compact, epithelial morphology while cells exposed to IR were overall less compact and appeared elongated and/or stretched. On the other hand, irradiated cells that were treated with hTERT-immortalized MSC EVs more closely resembled the control cells in their relative size and appearance, whereas irradiated cells that were treated with PC3 EVs appeared slightly larger and less compact. These images suggest that after multiple treatments, irradiated cells that received hTERT-immortalized MSC EVs regained and maintained their characteristic morphology and phenotype. These images also provide visual evidence that hTERT-immortalized MSC EVs may regulate cell cycle dynamics in damaged cells, which further supports the data in Figure 4b.

Given these results, we next assessed the relative expression of other cellular proteins that are associated with cell migration and/or reparative properties. The results in Figure 4d show that exposure of RPE cells to IR reduced the expression of N-cadherin, E-cadherin, DNA polymerase β, and VEGF and that the addition of hTERT-immortalized MSC EVs reversed this effect. Conversely, the exposure of RPE cells led to increased expression of CD142, and treatment with hTERT-immortalized MSC EVs lowered its expression to a level that was similar to the control cells. Lastly, we also observed a reduction in the expression of p-Akt in RPE cells that were exposed to IR, and, again, the addition of hTERT-immortalized MSC EVs restored its expression to a level that was similar to the control cells. Overall, the effect of PC3 EVs on the expression of each of these proteins was variable and not as robust as hTERT-immortalized MSC EVs. Collectively, these data indicate that multiple doses of hTERT-immortalized MSC EVs are capable of rescuing the expression of key proteins that are relevant for cell migration and repair in irradiated RPE cells.

### 3.5. Cargo Profiling of hTERT-Immortalized MSC EVs Reveals Proteins, Cytokines, and mRNAs Involved in Cellular Repair 

**Figure 5:**
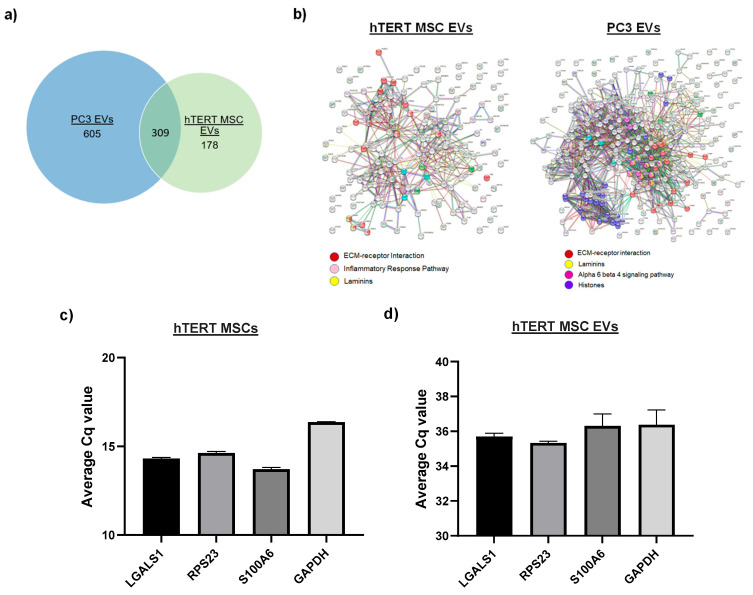

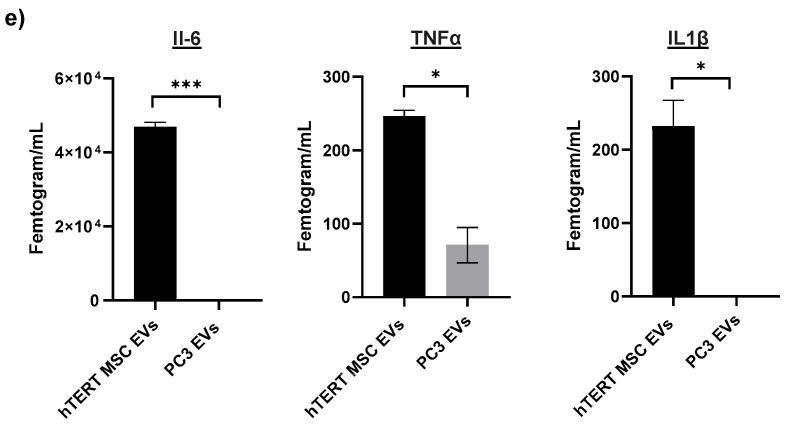
Profiling of EV-associated cargo. (**a**) The results from MS show the number of unique and shared proteins between hTERT-immortalized MSC EVs and PC3 EVs. (**b**) The STRING database was used to calculate the PPIs of peptides extracted from each EV preparation. Proteins are represented by nodes that are connected by lines representative of their confidence level. Colors represent specific functions that are associated with proteins. (**c**) Total RNA was isolated from hTERT-immortalized MSCs, and RT-qPCR was performed to target candidate mRNAs. Average Cq values are shown. *n* = 3. (**d**) Total RNA was isolated from hTERT-immortalized MSC EVs, and RT-qPCR was performed to target candidate mRNAs. Average Cq values are shown. *n* = 3. (**e**) EVs were assayed for the presence of inflammatory cytokines using the S-PLEX^®^ proinflammatory Panel 1 Kit (Meso Scale Diagnostics) following the manufacturer’s instructions. Assays were run in duplicate. Analyte concentrations were calculated using a 4-PL fit of a standard curve of the calibrators. The data shown represent samples assayed without lysis. *** *p* < 0.001; * *p* < 0.05.

The various types of EV-associated cargo are expected to drive functionality in recipient cells. While the literature characterizing the proteomic and RNA landscape of MSC EVs is growing, to the best of our knowledge, similar studies have not been performed on hTERT-immortalized MSC EVs. In an effort to screen EV-associated protein cargo, we first performed mass spectrometry. After processing the raw data, we identified approximately 605 and 178 proteins that were unique to PC3 EVs and hTERT-immortalized MSC EVs, respectively, and approximately 309 proteins that were common (Figure 5a). Proteins unique to hTERT-immortalized MSC EVs included peripherin and various isoforms of periostin, protein tyrosine phosphatase receptor gamma, and mannan-binding lectin serine protease 1, while proteins unique to PC3 EVs included moesin, ezrin, various isoforms of syntenin-1, ras-related proteins, annexins, and beta integrins. Proteins common among both EV preps include various isoforms of fibronectin, collagen, tenascin, and Target of Nesh-SH3. Protein interactions were further evaluated using the STRING database, and the resulting protein–protein interaction (PPI) networks are graphed in Figure 5b. Interestingly, the PPI networks of hTERT-immortalized MSC EVs and PC3 EVs both revealed nodes of proteins associated with laminins and ECM-receptor interactions, whereas protein nodes relating to inflammatory response and alpha 6 beta 4 signaling pathways were unique to hTERT-immortalized MSC EVs and PC3 EVs, respectively. 

We next assayed EVs for the presence of select mRNAs. Our rationale for these experiments is based on emerging evidence that EVs may serve as natural carriers of functional mRNA that can be delivered to recipient cells. For our initial screening, we consulted the RNAseq data from our previous experiments [70], which identified certain mRNA transcripts in stem cell EVs. From this list, we chose three candidate mRNAs (*LGALS1*, *RPS23*, *S100A6*) for confirmation by RT-qPCR. *GAPDH* was included primarily as a control for hTERT-immortalized MSC donor cells. After isolating total RNA from hTERT-immortalized MSC donor cells and hTERT-immortalized MSC EVs, RNA samples were enriched for mRNA (to exclude the possibility of non-functioning fragments) using Oligo (dT) magnetic beads. cDNA was generated using oligo (dT) primers. From there, we synthesized primers for our target transcripts and carried out qPCR using a SYBR^®^ Green protocol. As shown in Figure 5c, *LGALS1*, *RPS23*, and *S100A6* all displayed average Cq values that were lower than the average Cq value of *GAPDH* in hTERT-immortalized MSC donor cells, confirming that they were each present in relatively higher abundance than the control. Remarkably, we also detected each of these mRNAs in hTERT-immortalized MSC EVs at levels that were lower or similar to *GAPDH* (Figure 5d). These results support the presence of potential full-length mRNA within EVs. Whether these mRNAs are functional and are transferred to recipient cells remains to be studied.

For our last set of experiments, we screened for potential EV-associated cytokines. For these experiments, EVs were assayed against a panel of cytokines using a multiplex immunoassay platform. In the absence of lysis buffer, IL-6, TNFα, and IL1β were detected among hTERT-immortalized MSC EV samples; however, only TNFα was detected among PC3 EV samples. The relative concentrations of these cytokines are shown in Figure 5e. When samples were assayed in the presence of lysis buffer, the only detectable cytokine was IL-6 among the hTERT-immortalized MSC EV samples. The authors acknowledge that the observed expression of EV-associated cytokines is based on an input of standardized volume, and further confirmations can be performed by standardizing sample input on other parameters such as EV number or EV protein. Collectively, our cargo profiling highlights the differences that exist between stem cell and cancer EVs and identifies certain cargoes that may collectively contribute to the functional and reparative effects of hTERT-immortalized MSC EVs.

## 4. Discussion

Stem cell EVs represent a relatively novel and unique form of cell-free therapy. The literature supporting their potential therapeutic applications across multiple tissue types and pathologies is growing, as recently reviewed by others [23,24,71,72]. One area that has only recently begun to attract increased attention is the therapeutic potential of MSC EVs for ocular repair. The significant morbidity and economic burdens associated with vision loss and eye disease underscore the importance of novel treatment strategies.

To date, most of the literature has focused on EVs isolated from primary MSCs (e.g., bone marrow, adipose, umbilical). MSCs have been regarded as ideal sources for regenerative medicine because of their abundance/availability, multi-lineage differentiation potential, and vast reparative properties [73]. Despite these benefits, there are inherent challenges when working with primary MSCs. These include donor-to-donor variability in isolated tissues/cells and passage limitations due to their finite lifespan in culture. However, in 2009, Wolbank et al. reported the generation of telomerase-immortalized human adipose MSCs in an effort to overcome the limitations described above [74]. Since then, this breakthrough technology has been applied to advance the field of stem cell biology. Despite this success, the literature is conflicting regarding the possibility of hTERT-immortalized MSCs to harbor cancerous or malignant phenotypes [43,44,45]. It is therefore important to evaluate carefully the potential long-term complications that may be associated with both hTERT-immortalized MSCs and their EV byproducts if they are going to be considered for potential therapeutic use. For reference, the hTERT-immortalized MSC cells that were used in this study previously underwent karyology testing and were reported to exhibit a normal karyotype, suggesting that transduction by hTERT did not induce chromosomal abnormalities [74]. It is also worth pointing out that this rationale also holds true for EVs isolated from primary MSCs, as some researchers have observed pro-tumorigenic effects associated with their use [75,76,77,78]. While others have begun to assess the immunomodulatory properties of hTERT-immortalized MSCs [79,80], to the best of our knowledge, this is the first study to investigate the reparative effects of hTERT-immortalized MSC EVs in the context of eye repair.

The development of scalable and standardized platforms is an important consideration for the future of EV manufacturing, particularly for EVs with potential therapeutic applications such as stem cell EVs. EVs isolated from large-scale bulk volume offer several advantages including reduced lot-to-lot variability and improved reproducibility in downstream assays. In this study, we demonstrated both the scalability and reproducibility of our EV isolation platform by providing data generated from independent large-scale batches of EVs from hTERT-immortalized MSC cells and cancer (e.g., PC3, HCT 116) cells. Based on the NTA size distribution profiles, the EVs used in this study are expected to be a mixture of small EVs and potentially other smaller extracellular particles such as exomeres [81]. Our studies thus far have not resolved which of these EV subpopulations may be responsible for driving the observed functional effects. Future research will include additional EV purification steps to begin to address these questions.

Our characterization data highlight the different phenotypic and biochemical differences between hTERT-immortalized MSC EVs and cancer EVs. For example, EVs from cancer cells typically include subpopulations of larger vesicles (i.e., greater than 200 nm) relative to hTERT-immortalized MSC EVs. This may be attributed to the fact that cancer cells display more heterogeneity and, thus, produce and secrete even more heterogeneous EVs than non-cancerous cells, as suggested by others [82,83,84]. Although it is true that by their nature, EVs from all cell types exhibit a certain degree of heterogeneity, our data indicate a relative consistency in the distinct biochemical cargos of hTERT-immortalized MSC EVs vs. cancer (i.e., PC3, HCT 116) EVs. For example, we observed significant differences in the pattern of tetraspanin expression (e.g., CD63, CD81, CD9) when comparing hTERT-immortalized MSC EVs to both PC3 and HCT 116 EVs. Across two biological replicates each, hTERT-immortalized MSC EVs were most enriched with CD63, followed by CD81, and CD9; this pattern of expression was reversed in PC3 EVs and HCT 116 EVs. Interestingly, some of the literature suggests that high levels of CD9, which we found to be enriched in cancer EVs, have been correlated with increased metastasis in various cancers [85,86,87]. On the other hand, CD63, which we found to be enriched in hTERT-immortalized MSC EVs, has been associated with tumor suppression and decreased invasiveness [88,89,90].

The profiling of other EV-associated surface markers further revealed distinct differences between hTERT-immortalized MSC EVs and cancer EVs. Although the study of these markers remains relatively unexplored compared with other types of EV-associated cargo, their co-localization on the EV surface implies direct roles in various biological functions such as antigen presentation, uptake, and disease pathogenesis [52]. Our data highlighted a significant enrichment of certain cellular adhesion molecules, including MCAM, EpCAM, ICAM-1, EGFR, and ALCAM, in cancer EVs relative to hTERT-immortalized MSC EVs. Not surprisingly, each of these molecules is regarded as a marker of tumor progression and invasion when their levels are overexpressed [58,91,92,93,94]. Conversely, the molecules that were found to be more highly enriched in hTERT-immortalized MSC EVs (i.e., THY-1, Thrombomodulin, Neprilysin) have been associated with reparative, tumor suppressive, and anti-inflammatory properties [62,63,64,95,96,97].

For these reasons, we hypothesized that even if hTERT-immortalized MSC EVs and PC3 EVs exhibit similar superficial phenotypic effects that may appear to be reparative in nature, there may be less desirable and potentially irreparable effects on the underlying molecular and biochemical pathways in cells treated with cancer EVs. To some degree, this premise may be supported when comparing the results from the in vitro cell migration assay and the in vivo wound healing assay. Here, we found that a single fixed dose of hTERT-immortalized MSC EVs and PC3 EVs exerted comparable pro-migratory effects on RPE cells in vitro over the course of 48 h. Conversely, multiple subcutaneous injections of either hTERT-immortalized MSC EVs or PC3 EVs across multiple days yielded contrasting results in a mouse model of skin repair. Only high doses of hTERT-immortalized MSC EVs were associated with a significant reduction in the wound diameter after 6 and 9 days, whereas high doses of PC3 EVs had no substantial effect on reducing the wound diameter regardless of time. While the topic of skin repair is not directly related to eye repair, we believe that the results from the wound healing experiment demonstrate proof of concept for future in vivo studies relating to hTERT-immortalized MSC EV-mediated repair.

In our in vitro model of irradiation-induced damage, we found that a single dose of hTERT-immortalized MSC EVs was sufficient in restoring the viability of RPE cells, whereas the same dose of PC3 EVs had no impact on viability as measured by an ATP-based assay. Since exposure to IR is expected to activate the intrinsic pathway of apoptosis [98], we assessed the expression of Caspase 3 in EV-treated cells before and after IR. The addition of hTERT-immortalized MSC EVs restored the expression of uncleaved Caspase 3, consistent with the anti-apoptotic properties that we have previously observed with primary MSC EVs [68]. Our model of IR-induced damage also allowed us to examine the effects of EVs on cell cycle dynamics, as exposure to IR is well known for initiating cell cycle arrest at the G1 and G2 checkpoints [99]. The results from our study provide experimental evidence that hTERT-immortalized MSC EVs may primarily regulate the cell cycle at the G1/S transition, as indicated by the restoration of expression of Cyclin A, Cyclin D1, and Cyclin E after exposure to IR. This could partially explain why these EVs are effective in restoring the viability of damaged cells as they prevent premature entry into the S phase and permit proper response to DNA damage. Future studies will further elaborate on this concept by evaluating the effects of hTERT-immortalized MSC EVs on the regulatory and effector proteins responsible for controlling the DNA damage response, such as ATR, ATM, DNA-PK, and p53.

The proteins E-cadherin, N-cadherin, VEGF, and DNA polymerase β also play roles in cellular repair/homeostasis and/or DNA damage response [100,101,102,103]. Conversely, aberrant expression of E-cadherin, N-cadherin, and VEGF is also associated with transformation, metastasis, and tumor progression [104,105]. We, therefore, asked whether or not the addition of repetitive EV treatments to RPE cells would restore the relative expression levels of these proteins after exposure to irradiation, which would be consistent with the initiation of reparative processes. We were also interested in understanding whether EV treatments would result in a collective overexpression of these proteins relative to the control RPEs, which could possibly point toward a loss of cellular polarization and induction of cellular transformation. This is an important issue that needs to be resolved when considering any EV-based treatment as any potential dysregulation of repair processes could promote an environment that would be favorable for cancer formation.

Indeed, we found that IR caused a drastic reduction in the expression of E-cadherin, N-cadherin, VEGF, and DNA polymerase β and that hTERT-immortalized MSC EVs were able to partially restore the expression of these proteins. Importantly, when compared to the control, the expression levels of these proteins displayed relatively similar expression compared to control RPEs. We also found that the effect of PC3 EVs on the expression of these proteins was variable and not as robust as hTERT-immortalized MSC EVs. We acknowledge that the protein expression levels that we observed in response to EV treatment may only be temporary or transient in nature. To better examine potential long-term effects, a soft agar assay could be performed to assess whether hTERT-immortalized MSC EVs may cause cells to lose their anchorage dependence and promote colony formation, which would be indicative of cellular transformation. While outside of the scope of the current manuscript, future studies should further evaluate this topic to rule out any potential carcinogenicity associated with hTERT-immortalized MSC EVs.

The results highlighted above are consistent with the existing literature describing the anti-apoptotic and pro-reparative properties of EVs isolated from primary/non-immortalized MSCs. Previous data from others suggest that these effects could be the result of EV-mediated activation of the Akt signaling pathway [106,107,108,109,110]. Our data also point toward a potential hTERT-immortalized MSC EV-modulated activation of Akt in damaged RPE cells, as indicated by the increased expression of p-Akt in response to EV treatment. It is worth noting that although PC3 EVs exhibited similar effects in restoring certain protein levels, the authors do not advocate the use of cancer EVs for reparative purposes, and further interpretation of these data is outside of the scope of the current study. 

At this point, we believe EV-mediated reparative effects may be collectively driven by multiple different cargoes, including surface-associated and EV-encapsulated proteins, cytokines, and RNAs. To that end, we recently performed preliminary studies to see whether the inactivation of EV-associated proteins and/or RNAs would diminish their functional effects. Using the same model of IR-induced damage, we treated irradiated RPEs with EVs that had been treated with either heat to inactivate EV-associated protein cargo or Ultraviolet-C (UV-C) light to inactive EV-associated RNA cargo and then performed a cell viability assay. Not surprisingly, the results from this experiment showed treatment of hTERT-immortalized MSC EVs with both heat and UV-C significantly decreased their capacity to restore viability compared with non-treated EVs (Figure S5a). This implies that both EV-associated RNA and protein content play critical roles in mediating functional effects. Interestingly, there were no appreciable effects when the same study was carried out using PC3 EVs (Figure S5b). This may be due to the fact that PC3 EVs are much more enriched with molecular cargo that already overwhelms recipient cells upon uptake; this notion is somewhat supported by the fact that untreated PC3 EVs led to an even further reduction in the viability of RPE cells.

The results from mass spectrometry and the corresponding PPI networks further highlight the differences between hTERT-immortalized MSC EVs and PC3 EVs. As expected, PC3 EVs had a higher number of unique proteins and much more overlap and redundancy in their protein networks compared with hTERT-immortalized MSC EVs. A somewhat surprising finding in the current study was the detection of certain inflammatory cytokines that were present in significantly higher concentrations in hTERT-immortalized MSC EV samples relative to PC3 EV samples. These cytokines, namely, IL-6, TNFα, and IL1β, are regarded as pleotropic cytokines with effects ranging from inflammation and immunoregulation to apoptosis, DNA repair, and cell proliferation [111,112,113,114,115,116]. For this reason, we speculate that EV-associated cytokines could act both directly on damaged cells and indirectly by further recruiting innate immune cells to the damaged environment. In this sense, EV-associated cytokines may augment the ability of the host cell to regulate the inflammatory response to injury and/or damage. While the current data do not suggest that these cytokines are encapsulated within the EV membrane, whether or not they are membrane-bound or co-isolates that remain after EV isolation remains to be further studied.

In the current study, we also present data to support the relatively novel concept of EV-associated mRNAs. The concept of selectively loading EVs with synthetic mRNAs has been discussed as an improved strategy for drug delivery or vaccine development, as recently reviewed elsewhere [117,118]; however, the screening of EV-associated mRNAs remains relatively understudied relative to other types of RNAs. For our initial screening, we consulted the RNAseq data from our previous experiments [68]. From there, we selected candidate mRNAs based on their relevance to cellular repair and their relatively high abundance in primary MSCs. After confirming their presence in donor hTERT-immortalized MSC cells, we then confirmed their association with hTERT-immortalized MSC EVs. Although the present study does not directly address whether these mRNAs are effectively delivered to recipient cells and, if so, whether they are translated into functional proteins, it nonetheless provides proof of concept for the EV-mediated delivery of natural mRNAs to target cells. Follow-up studies are warranted to further explore this subject. 

It has been documented that exposure to radiation can lead to several ocular complications including, but not limited to, those affecting the cornea, conjunctiva, iris, retina, and optic nerve [119]. Previous reports have also documented the occupational hazards and ophthalmic consequences (i.e., cataracts, lens damage) that are associated with exposure to ionizing radiation during or after radiation therapy [120,121]. Therefore, the data presented here highlights a relevant EV-based platform that could be applied to reduce or reverse radiation-induced ocular damage. These results could also potentially translate to other models of eye-related damage and EV-mediated eye repair. To the best of our knowledge, this is the first study to establish proof of concept for reproducible, large-scale manufacturing of hTERT-immortalized MSC EVs and to investigate their reparative properties in vitro using RPE cells. For this reason, hTERT-immortalized MSCs may represent a more consistent platform than primary MSCs for generating functional EVs for downstream therapeutic evaluation. However, as mentioned above, further testing will be required to evaluate the long-term safety profile and any associated side effects that could be associated with these EVs. Collectively, this research sets a precedent for continued efforts to investigate novel applications of hTERT-immortalized MSC EVs not only for eye repair but for any cell type exhibiting a damaged or diseased phenotype.

## Figures and Tables

**Table 1:** List of primary antibodies.

Antibody	Vendor	Catalog
α-CD81	SBI (Palo Alto, CA, USA)	EXOAB-CD81A-1
α-CD63	SBI	EXOAB-CD63A-1
α-CD9	SBI	EXOAB-CD9A-1
α-GAPDH	Santa Cruz Biotechnology (Dallas, TX, USA)	sc-47724
α-Caspase 3	Santa Cruz Biotechnology	sc-7148
α-Cyclin A	Santa Cruz Biotechnology	sc-751
α-Cyclin D1	Santa Cruz Biotechnology	sc-753
α-Cyclin E	Santa Cruz Biotechnology	sc-198
α-Actin	Santa Cruz Biotechnology	sc-8432
α-N-cadherin	Cell Signaling Technology (Danvers, MA, USA)	14215S
α-E-cadherin	Cell Signaling Technology	3195T
α-CD142	Cell Signaling Technology	97438T
α-VEGF	Santa Cruz Biotechnology	sc-57496
α-DNA Polymerase β	Novus Biologicals (Centennial, CO, USA)	NBP2-38600
α-p-AKT	Santa Cruz Biotechnology	sc-16646-R
α-AKT	Cell Signaling Technology	9272S

**Table 2:** Primer sequences and annealing temperatures (Tm).

Primer	Forward Sequence	Reverse Sequence	Tm
S100A6	5’-GTGACAAGCACACCCTGAGCAA-3’	5’-GGAAGTTCACCTCCTGGTCCTT-3’	58.1 °C
RPS23	5’-AGGAAGTGT GTAAGGGTCCAGC-3’	5’-CACCAACAGCATGACCTTTGCG-3’	58.9 °C
LGALS1	5’-AGCAGCGGGAGGCTGTCTTTC-3’	5’-ATCCATCTGGCAGCTTGACGGT-3′	61.2 °C
GAPDH	5’-GAAGGTGAAGGTCGG AGTCAAC-3’	5’-CAGAGTTAAAAGCAGCCCTGGT-3’	57.5 °C

## Data Availability

The proteomics data files were deposited to the ProteomeXchange Consortium via the PRIDE partner repository with the dataset identifier PXD044629.

## Supplementary Materials

The following supporting information can be downloaded at: https://www.mdpi.com/article/10.3390/cells13100861/s1, Figure S1. Full-length Western blot images; Figure S2. Nanoparticle tracking analysis and characterization of HCT 116 EVs; Figure S3. Surface marker profiling of HCT 116 EVs; Figure S4. In vivo wound healing assay; Figure S5. The effect of inactivated EVs on irradiated RPEs. Table S1. Average ECL values of EV-associated surface markers.

## References

[1] Trounson A., McDonald C. (2015). Stem Cell Therapies in Clinical Trials: Progress and Challenges. Cell Stem Cell.

[2] Andrzejewska A., Lukomska B., Janowsk M. (2019). Concise Review: Mesenchymal Stem Cells: From Roots to Boost. Stem Cells.

[3] Sengupta V., Sengupta S., Lazo A., Woods P., Nolan A., Bremer N. (2020). Exosomes Derived from Bone Marrow Mesenchymal Stem Cells as Treatment for Severe COVID-19. Stem Cells Dev..

[4] Saburi E., Abazari M.F., Hassannia H., Mansour R.N., Eshaghi-Gorji R., Gheibi M., Rahmati M., Enderami S.E. (2021). The use of mesenchymal stem cells in the process of treatment and tissue regeneration after recovery in patients with COVID-19. Gene.

[5] Shi L., Wang L., Xu R., Zhang C., Xie Y., Liu K., Li T., Hu W., Zhen C., Wang F.S. (2021). Mesenchymal stem cell therapy for severe COVID-19. Signal Transduct. Target. Ther..

[6] Zhu R., Yan T., Feng Y., Liu Y., Cao H., Peng G., Yang Y., Xu Z., Liu J., Hou W. (2021). Mesenchymal stem cell treatment improves outcome of COVID-19 patients via multiple immunomodulatory mechanisms. Cell Res..

[7] Liang X., Ding Y., Zhang Y., Tse H.F., Lian Q. (2014). Paracrine mechanisms of mesenchymal stem cell-based therapy: Current status and perspectives. Cell Transplant..

[8] Fan X.L., Zhang Y., Li X., Fu Q.L. (2020). Mechanisms underlying the protective effects of mesenchymal stem cell-based therapy. Cell. Mol. Life Sci..

[9] Maacha S., Sidahmed H., Jacob S., Gentilcore G., Calzone R., Grivel J.C., Cugno C. (2020). Paracrine Mechanisms of Mesenchymal Stromal Cells in Angiogenesis. Stem Cells Int..

[10] Buzas E.I. (2023). The roles of extracellular vesicles in the immune system. Nat. Rev. Immunol..

[11] Keshtkar S., Azarpira N., Ghahremani M.H. (2018). Mesenchymal stem cell-derived extracellular vesicles: Novel frontiers in regenerative medicine. Stem Cell Res. Ther..

[12] Hur Y.H., Cerione R.A., Antonyak M.A. (2020). Extracellular vesicles and their roles in stem cell biology. Stem Cells.

[13] Welsh J.A., Goberdhan D.C.I., O’Driscoll L., Buzas E.I., Blenkiron C., Bussolati B., Cai H., Di Vizio D., Driedonks T.A.P., Erdbrügger U. (2024). Minimal information for studies of extracellular vesicles (MISEV2023): From basic to advanced approaches. J. Extracell. Vesicles.

[14] Ferguson S.W., Wang J., Lee C.J., Liu M., Neelamegham S., Canty J.M., Nguyen J. (2018). The microRNA regulatory landscape of MSC-derived exosomes: A systems view. Sci. Rep..

[15] Branscome H., Paul S., Yin D., El-Hage N., Agbottah E.T., Zadeh M.A., Liotta L.A., Kashanchi F. (2020). Use of Stem Cell Extracellular Vesicles as a “Holistic” Approach to CNS Repair. Front. Cell Dev. Biol..

[16] Roefs M.T., Sluijter J.P.G., Vader P. (2020). Extracellular Vesicle-Associated Proteins in Tissue Repair. Trends Cell Biol..

[17] Wang L., Hu L., Zhou X., Xiong Z., Zhang C., Shehada H.M.A., Hu B., Song J., Chen L. (2017). Exosomes secreted by human adipose mesenchymal stem cells promote scarless cutaneous repair by regulating extracellular matrix remodelling. Sci. Rep..

[18] Xin H., Katakowski M., Wang F., Qian J.Y., Liu X.S., Ali M.M., Buller B., Zhang Z.G., Chopp M. (2017). MicroRNA cluster miR-17-92 Cluster in Exosomes Enhance Neuroplasticity and Functional Recovery after Stroke in Rats. Stroke.

[19] Liu L., Jin X., Hu C.F., Li R., Zhou Z., Shen C.X. (2017). Exosomes Derived from Mesenchymal Stem Cells Rescue Myocardial Ischaemia/Reperfusion Injury by Inducing Cardiomyocyte Autophagy Via AMPK and Akt Pathways. Cell Physiol Biochem.

[20] Wang L., Pei S., Han L., Guo B., Li Y., Duan R., Yao Y., Xue B., Chen X., Jia Y. (2018). Mesenchymal Stem Cell-Derived Exosomes Reduce A1 Astrocytes via Downregulation of Phosphorylated NFκB P65 Subunit in Spinal Cord Injury. Cell Physiol. Biochem..

[21] Feng N., Jia Y., Huang X. (2019). Exosomes from adipose-derived stem cells alleviate neural injury caused by microglia activation via suppressing NF-kB and MAPK pathway. J. Neuroimmunol..

[22] Ren S., Chen J., Duscher D., Liu Y., Guo G., Kang Y., Xiong H., Zhan P., Wang Y., Wang C. (2019). Microvesicles from human adipose stem cells promote wound healing by optimizing cellular functions via AKT and ERK signaling pathways. Stem Cell Res. Ther..

[23] Dabrowska S., Andrzejewska A., Janowski M., Lukomska B. (2021). Immunomodulatory and Regenerative Effects of Mesenchymal Stem Cells and Extracellular Vesicles: Therapeutic Outlook for Inflammatory and Degenerative Diseases. Front. Immunol..

[24] Kou M., Huang L., Yang J., Chiang Z., Chen S., Liu J., Guo L., Zhang X., Zhou X., Xu X. (2022). Mesenchymal stem cell-derived extracellular vesicles for immunomodulation and regeneration: A next generation therapeutic tool?. Cell Death Dis..

[25] Marques A.P., Ramke J., Cairns J., Butt T., Zhang J.H., Jones I., Jovic M., Nandakumar A., Faal H., Taylor H. (2022). The Economics of Vision Impairment and it’s Leading Causes: A Systematic Review. EClinicalMedicine.

[26] Rein D.B., Wittenborn J.S., Zhang P., Sublett F., Lamuda P.A., Lundeen E.A., Saaddine J. (2022). The Economic Burden of Vision Loss and Blindness in the United States. Ophthalmology.

[27] Sia R.K., Ryan D.S., Brooks D.I., Kagemann J.M., Bower K.S., French L.M., Justin G.A., Colyer M.H. (2022). The Impact of Combat Ocular Trauma and Traumatic Brain Injury on Vision- and Health-Related Quality of Life among U.S. Military Casualties. Mil. Med..

[28] Joyce N.C., Harris D.L., Markov V., Zhang Z., Saitta B. (2012). Potential of human umbilical cord blood mesenchymal stem cells to heal damaged corneal endothelium. Mol. Vis..

[29] Mead B., Berry M., Logan A., Scott R.A., Leadbeater W., Scheven B.A. (2015). Stem cell treatment of degenerative eye disease. Stem Cell Res..

[30] Adak S., Magdalene D., Deshmukh S., Das D., Jaganathan B.G. (2021). A Review on Mesenchymal Stem Cells for Treatment of Retinal Diseases. Stem Cell Rev. Rep..

[31] Harrell C.R., Simovic M.B., Fellabaum C., Arsenijevic A., Djonov V., Arsenijevic N., Volarevic V. (2018). Therapeutic Potential of Mesenchymal Stem Cell-Derived Exosomes in the Treatment of Eye Diseases. Adv. Exp. Med. Biol..

[32] Nuzzi R., Caselgrandi P., Vercelli A. (2020). Effect of Mesenchymal Stem Cell-Derived Exosomes on Retinal Injury: A Review of Current Findings. Stem Cells Int..

[33] Bonnell E., Pasquier E., Wellinger R.J. (2021). Telomere Replication: Solving Multiple End Replication Problems. Front. Cell Dev. Biol..

[34] Parsch D., Fellenberg J., Brümmendorf T.H., Eschlbeck A.M., Richter W. (2004). Telomere length and telomerase activity during expansion and differentiation of human mesenchymal stem cells and chondrocytes. J. Mol. Med..

[35] Bernardo M.E., Zaffaroni N., Novara F., Cometa A.M., Avanzini M.A., Moretta A., Montagna D., Maccario R., Villa R., Daidone M.G. (2007). Human bone marrow derived mesenchymal stem cells do not undergo transformation after long-term in vitro culture and do not exhibit telomere maintenance mechanisms. Cancer Res..

[36] Zimmermann S., Voss M., Kaiser S., Kapp U., Waller C.F., Martens U.M. (2003). Lack of telomerase activity in human mesenchymal stem cells. Leukemia.

[37] Ouellette M.M., McDaniel L.D., Wright W.E., Shay J.W., Schultz R.A. (2000). The establishment of telomerase-immortalized cell lines representing human chromosome instability syndromes. Hum. Mol. Genet..

[38] Kassem M., Abdallah B.M., Yu Z., Ditzel N., Burns J.S. (2004). The use of hTERT-immortalized cells in tissue engineering. Cytotechnology.

[39] Belair C.D., Yeager T.R., Lopez P.M., Reznikoff C.A. (1997). Telomerase activity: A biomarker of cell proliferation, not malignant transformation. Proc. Natl. Acad. Sci. USA.

[40] Lee K.M., Choi K.H., Ouellette M.M. (2004). Use of exogenous hTERT to immortalize primary human cells. Cytotechnology.

[41] Liu T.M., Ng W.M., Tan H.S., Vinitha D., Yang Z., Fan J.B., Zou Y., Hui J.H., Lee E.H., Lim B. (2013). Molecular basis of immortalization of human mesenchymal stem cells by combination of p53 knockdown and human telomerase reverse transcriptase overexpression. Stem Cells Dev..

[42] James S., Fox J., Afsari F., Lee J., Clough S., Knight C., Ashmore J., Ashton P., Preham O., Hoogduijn M. (2015). Multiparameter Analysis of Human Bone Marrow Stromal Cells Identifies Distinct Immunomodulatory and Differentiation-Competent Subtypes. Stem Cell Rep..

[43] Siska E.K., Weisman I., Romano J., Ivics Z., Izsvák Z., Barkai U., Petrakis S., Koliakos G. (2017). Generation of an immortalized mesenchymal stem cell line producing a secreted biosensor protein for glucose monitoring. PLoS ONE.

[44] Milyavsky M., Shats I., Erez N., Tang X., Senderovich S., Meerson A., Tabach Y., Goldfinger N., Ginsberg D., Harris C.C. (2003). Prolonged culture of telomerase-immortalized human fibroblasts leads to a premalignant phenotype. Cancer Res..

[45] Park Y.J., Kim E.K., Bae J.Y., Moon S., Kim J. (2016). Human telomerase reverse transcriptase (hTERT) promotes cancer invasion by modulating cathepsin D via early growth response (EGR)-1. Cancer Lett..

[46] Bikkul M.U., Faragher R.G.A., Worthington G., Meinke P., Kerr A.R.W., Sammy A., Riyahi K., Horton D., Schirmer E.C., Hubank M. (2019). Telomere elongation through hTERT immortalization leads to chromosome repositioning in control cells and genomic instability in Hutchinson-Gilford progeria syndrome fibroblasts, expressing a novel SUN1 isoform. Genes Chromosomes Cancer.

[47] Branscome H., Paul S., Khatkar P., Kim Y., Barclay R.A., Pinto D.O., Yin D., Zhou W., Liotta L.A., El-Hage N. (2020). Stem Cell Extracellular Vesicles and their Potential to Contribute to the Repair of Damaged CNS Cells. J. Neuroimmune Pharmacol..

[48] Howard M., Erickson J., Cuba Z., Kim S., Zhou W., Gade P., Carter R., Mitchell K., Branscome H., Siddhi D. (2022). A secretory form of Parkin-independent mitophagy contributes to the repertoire of extracellular vesicles released into the tumour interstitial fluid in vivo. J. Extracell. Vesicles.

[49] Heberle H., Meirelles G.V., da Silva F.R., Telles G.P., Minghim R. (2015). InteractiVenn: A web-based tool for the analysis of sets through Venn diagrams. BMC Bioinform..

[50] Jaccard N., Griffin L.D., Keser A., Macown R.J., Super A., Veraitch F.S., Szita N. (2014). Automated method for the rapid and precise estimation of adherent cell culture characteristics from phase contrast microscopy images. Biotechnol. Bioeng..

[51] Hallal S., Tűzesi Á., Grau G.E., Buckland M.E., Alexander K.L. (2022). Understanding the extracellular vesicle surface for clinical molecular biology. J. Extracell. Vesicles.

[52] Lam K.C.K., Lam M.K.N., Chim C.S., Chan G.C.F., Li J.C.B. (2020). The functional role of surface molecules on extracellular vesicles in cancer, autoimmune diseases, and coagulopathy. J. Leucoc. Biol..

[53] Roland C.L., Harken A.H., Sarr M.G., Barnett C.C. (2007). ICAM-1 expression determines malignant potential of cancer. Surgery.

[54] Spizzo G., Fong D., Wurm M., Ensinger C., Obrist P., Hofer C., Mazzoleni G., Gastl G., Went P. (2011). EpCAM expression in primary tumour tissues and metastases: An immunohistochemical analysis. J. Clin. Pathol..

[55] Wu Z., Wu Z., Li J., Yang X., Wang Y., Yu Y., Ye J., Xu C., Qin W., Zhang Z. (2012). MCAM is a novel metastasis marker and regulates spreading, apoptosis and invasion of ovarian cancer cells. Tumor Biol..

[56] Sasaki T., Hiroki K., Yamashita Y. (2013). The role of epidermal growth factor receptor in cancer metastasis and microenvironment. BioMed Res. Int..

[57] Wang M.H., Sun R., Zhou X.M., Zhang M.Y., Lu J.B., Yang Y., Zeng L.S., Yang X.Z., Shi L., Xiao R.W. (2018). Epithelial cell adhesion molecule overexpression regulates epithelial-mesenchymal transition, stemness and metastasis of nasopharyngeal carcinoma cells via the PTEN/AKT/mTOR pathway. Cell Death Dis..

[58] Darvishi B., Boroumandieh S., Majidzadeh A.K., Salehi M., Jafari F., Farahmand L. (2020). The role of activated leukocyte cell adhesion molecule (ALCAM) in cancer progression, invasion, metastasis and recurrence: A novel cancer stem cell marker and tumor-specific prognostic marker. Exp. Mol. Pathol..

[59] Uribe M.L., Marrocco I., Yarden Y. (2021). EGFR in Cancer: Signaling Mechanisms, Drugs, and Acquired Resistance. Cancers.

[60] Du X., Zhang Q., Wang S., Chen X., Wang Y. (2022). MCAM is associated with metastasis and poor prognosis in osteosarcoma by modulating tumor cell migration. J. Clin. Lab. Anal..

[61] Rege T.A., Hagood J.S. (2006). Thy-1 as a regulator of cell-cell and cell-matrix interactions in axon regeneration, apoptosis, adhesion, migration, cancer, and fibrosis. FASEB J..

[62] Loghmani H., Conway E.M. (2018). Exploring traditional and nontraditional roles for thrombomodulin. Blood.

[63] Huang T.C., Wu H.L., Chen S.H., Wang Y.T., Wu C.C. (2020). Thrombomodulin facilitates peripheral nerve regeneration through regulating M1/M2 switching. J. Neuroinflamm..

[64] Nalivaeva N.N., Zhuravin I.A., Turner A.J. (2020). Neprilysin expression and functions in development, ageing and disease. Mech. Ageing Dev..

[65] Sankhe R., Pai S.R.K., Kishore A. (2021). Tumour suppression through modulation of neprilysin signaling: A comprehensive review. Eur. J. Pharmacol..

[66] Yang J., Zhan X.Z., Malola J., Li Z.Y., Pawar J.S., Zhang H.T., Zha Z.G. (2020). The multiple roles of Thy-1 in cell differentiation and regeneration. Differentiation.

[67] Sedov E., Koren E., Chopra S., Ankawa R., Yosefzon Y., Yusupova M., Weiss L.E., Mahly A., Soffer A., Feldman A. (2022). THY1-mediated mechanisms converge to drive YAP activation in skin homeostasis and repair. Nat. Cell Biol..

[68] Desouky O., Ding N., Zhou G. (2015). Targeted and non-targeted effects of ionizing radiation. J. Radiat. Res. Appl. Sci..

[69] Mavragani I.V., Laskaratou D.A., Frey B., Candéias S.M., Gaipl U.S., Lumniczky K., Georgakilas A.G. (2015). Key mechanisms involved in ionizing radiation-induced systemic effects. A current review. Toxicol. Res..

[70] Branscome H., Khatkar P., Al Sharif S., Yin D., Jacob S., Cowen M., Kim Y., Erickson J., Brantner C.A., El-Hage N. (2022). Retroviral infection of human neurospheres and use of stem Cell EVs to repair cellular damage. Sci. Rep..

[71] Öztürk S., Elçin A.E., Koca A., Elçin Y.M. (2021). Therapeutic Applications of Stem Cells and Extracellular Vesicles in Emergency Care: Futuristic Perspectives. Stem Cell Rev. Rep..

[72] Yin L., Liu X., Shi Y., Ocansey D.K.W., Hu Y., Li X., Zhang C., Xu W., Qian H. (2020). Therapeutic Advances of Stem Cell-Derived Extracellular Vesicles in Regenerative Medicine. Cells.

[73] Han Y., Li X., Zhang Y., Han Y., Chang F., Ding J. (2019). Mesenchymal Stem Cells for Regenerative Medicine. Cells.

[74] Wolbank S., Stadler G., Peterbauer A., Gillich A., Karbiener M., Streubel B., Wieser M., Katinger H., van Griensven M., Redl H. (2009). Telomerase immortalized human amnion- and adipose-derived mesenchymal stem cells: Maintenance of differentiation and immunomodulatory characteristics. Tissue Eng. Part A.

[75] Lin R., Wang S., Zhao R.C. (2013). Exosomes from human adipose-derived mesenchymal stem cells promote migration through Wnt signaling pathway in a breast cancer cell model. Mol. Cell. Biochem..

[76] Roccaro A.M., Sacco A., Maiso P., Azab A.K., Tai Y.T., Reagan M., Azab F., Flores L.M., Campigotto F., Weller E. (2013). BM mesenchymal stromal cell-derived exosomes facilitate multiple myeloma progression. J. Clin. Investig..

[77] Shi S., Zhang Q., Xia Y., You B., Shan Y., Bao L., Li L., You Y., Gu Z. (2016). Mesenchymal stem cell-derived exosomes facilitate nasopharyngeal carcinoma progression. Am. J. Cancer Res..

[78] Qin F., Tang H., Zhang Y., Zhang Z., Huang P., Zhu J. (2020). Bone marrow-derived mesenchymal stem cell-derived exosomal microRNA-208a promotes osteosarcoma cell proliferation, migration, and invasion. J. Cell Physiol..

[79] Gómez-Ferrer M., Villanueva-Badenas E., Sánchez-Sánchez R., Sánchez-López C.M., Baquero M.C., Sepúlveda P., Dorronsoro A. (2021). HIF-1α and Pro-Inflammatory Signaling Improves the Immunomodulatory Activity of MSC-Derived Extracellular Vesicles. Int. J. Mol. Sci..

[80] Haghighitalab A., Matin M.M., Amin A., Minaee S., Bidkhori H.R., Doeppner T.R., Bahrami A.R. (2021). Investigating the effects of IDO1, PTGS2, and TGF-β1 overexpression on immunomodulatory properties of hTERT-MSCs and their extracellular vesicles. Sci. Rep..

[81] Anand S., Samuel M., Mathivanan S. (2021). Exomeres: A New Member of Extracellular Vesicles Family. Subcell Biochem..

[82] Kalluri R. (2016). The biology and function of exosomes in cancer. J. Clin. Investig..

[83] Willms E., Cabañas C., Mäger I., Wood M.J.A., Vader P. (2018). Extracellular Vesicle Heterogeneity: Subpopulations, Isolation Techniques, and Diverse Functions in Cancer Progression. Front. Immunol..

[84] Wang S.E. (2020). Extracellular Vesicles and Metastasis. Cold Spring Harb. Perspect. Med..

[85] Kischel P., Bellahcene A., Deux B., Lamour V., Dobson R., DE Pauw E., Clezardin P., Castronovo V. (2012). Overexpression of CD9 in human breast cancer cells promotes the development of bone metastases. Anticancer. Res..

[86] Lorico A., Lorico-Rappa M., Karbanová J., Corbeil D., Pizzorno G. (2021). CD9, a tetraspanin target for cancer therapy?. Exp. Biol. Med..

[87] Nigri J., Leca J., Tubiana S.S., Finetti P., Guillaumond F., Martinez S., Lac S., Iovanna J.L., Audebert S., Camoin L. (2022). CD9 mediates the uptake of extracellular vesicles from cancer-associated fibroblasts that promote pancreatic cancer cell aggressiveness. Sci. Signal..

[88] Lupia A., Peppicelli S., Witort E., Bianchini F., Carloni V., Pimpinelli N., Urso C., Borgognoni L., Capaccioli S., Calorini L. (2014). CD63 tetraspanin is a negative driver of epithelial-to-mesenchymal transition in human melanoma cells. J. Investig. Dermatol..

[89] Liu W.H., Li X., Zhu X.L., Hou M.L., Zhao W. (2018). CD63 inhibits the cell migration and invasion ability of tongue squamous cell carcinoma. Oncol. Lett..

[90] Yu S., Chen J., Quan M., Li L., Li Y., Gao Y. (2021). CD63 negatively regulates hepatocellular carcinoma development through suppression of inflammatory cytokine-induced STAT3 activation. J. Cell. Mol. Med..

[91] Sigismund S., Avanzato D., Lanzetti L. (2018). Emerging functions of the EGFR in cancer. Mol. Oncol..

[92] An Y., Wei N., Cheng X., Li Y., Liu H., Wang J., Xu Z., Sun Z., Zhang X. (2020). MCAM abnormal expression and clinical outcome associations are highly cancer dependent as revealed through pan-cancer analysis. Brief. Bioinform..

[93] Bui T.M., Wiesolek H.L., Sumagin R. (2020). ICAM-1: A master regulator of cellular responses in inflammation, injury resolution, and tumorigenesis. J. Leucoc. Biol..

[94] Huang L., Yang Y., Yang F., Liu S., Zhu Z., Lei Z., Guo J. (2018). Functions of EpCAM in physiological processes and diseases (Review). Int. J. Mol. Med..

[95] Lee M.J., Shin J.O., Jung H.S. (2013). Thy-1 knockdown retards wound repair in mouse skin. J. Dermatol. Sci..

[96] Martin F.A., Murphy R.P., Cummins P.M. (2013). Thrombomodulin and the vascular endothelium: Insights into functional, regulatory, and therapeutic aspects. Am. J. Physiol. Heart Circ. Physiol..

[97] Saalbach A., Anderegg U. (2019). Thy-1: More than a marker for mesenchymal stromal cells. FASEB J..

[98] Cao X., Wen P., Fu Y., Gao Y., Qi X., Chen B., Tao Y., Wu L., Xu A., Lu H. (2019). Radiation induces apoptosis primarily through the intrinsic pathway in mammalian cells. Cell. Signal..

[99] Teyssier F., Bay J.O., Dionet C., Verrelle P. (1999). Régulation du cycle cellulaire des cellules exposées aux radiations ionisantes [Cell cycle regulation after exposure to ionizing radiation]. Bull. Cancer.

[100] Jones M., Sabatini P.J., Lee F.S., Bendeck M.P., Langille B.L. (2002). N-cadherin upregulation and function in response of smooth muscle cells to arterial injury. Arterioscler. Thromb. Vasc. Biol..

[101] Schnoor M. (2015). E-cadherin Is Important for the Maintenance of Intestinal Epithelial Homeostasis Under Basal and Inflammatory Conditions. Dig. Dis. Sci..

[102] Johnson K.E., Wilgus T.A. (2014). Vascular Endothelial Growth Factor and Angiogenesis in the Regulation of Cutaneous Wound Repair. Adv. Wound Care.

[103] Ray S., Menezes M.R., Senejani A., Sweasy J.B. (2013). Cellular roles of DNA polymerase beta. Yale J. Biol. Med..

[104] Loh C.Y., Chai J.Y., Tang T.F., Wong W.F., Sethi G., Shanmugam M.K., Chong P.P., Looi C.Y. (2019). The E-Cadherin and N-Cadherin Switch in Epithelial-to-Mesenchymal Transition: Signaling, Therapeutic Implications, and Challenges. Cells.

[105] Yang Y., Cao Y. (2022). The impact of VEGF on cancer metastasis and systemic disease. Semin Cancer Biol..

[106] Shabbir A., Cox A., Rodriguez-Menocal L., Salgado M., Van Badiavas E. (2015). Mesenchymal Stem Cell Exosomes Induce Proliferation and Migration of Normal and Chronic Wound Fibroblasts, and Enhance Angiogenesis In Vitro. Stem Cells Dev..

[107] Gu H., Ji R., Zhang X., Wang M., Zhu W., Qian H., Chen Y., Jiang P., Xu W. (2016). Exosomes derived from human mesenchymal stem cells promote gastric cancer cell growth and migration via the activation of the Akt pathway. Mol. Med. Rep..

[108] Pan Q., Wang Y., Lan Q., Wu W., Li Z., Ma X., Yu L. (2019). Exosomes Derived from Mesenchymal Stem Cells Ameliorate Hypoxia/Reoxygenation-Injured ECs via Transferring MicroRNA-126. Stem Cells Int..

[109] Qiu X., Liu J., Zheng C., Su Y., Bao L., Zhu B., Liu S., Wang L., Wang X., Wang Y. (2020). Exosomes released from educated mesenchymal stem cells accelerate cutaneous wound healing via promoting angiogenesis. Cell Prolif..

[110] Wei H., Xu Y., Chen Q., Chen H., Zhu X., Li Y. (2020). Mesenchymal stem cell-derived exosomal miR-223 regulates neuronal cell apoptosis. Cell Death Dis..

[111] Ernandez T., Mayadas T.N. (2009). Immunoregulatory role of TNFalpha in inflammatory kidney diseases. Kidney Int..

[112] Lopez-Castejon G., Brough D. (2011). Understanding the mechanism of IL-1β secretion. Cytokine Growth Factor Rev..

[113] Hewett S.J., Jackman N.A., Claycomb R.J. (2012). Interleukin-1β in Central Nervous System Injury and Repair. Eur. J. Neurodegener. Dis..

[114] Tachibana S., Zhang X., Ito K., Ota Y., Cameron A.M., Williams G.M., Sun Z. (2014). Interleukin-6 is required for cell cycle arrest and activation of DNA repair enzymes after partial hepatectomy in mice. Cell Biosci..

[115] Jang D.I., Lee A.H., Shin H.Y., Song H.R., Park J.H., Kang T.B., Lee S.R., Yang S.H. (2021). The Role of Tumor Necrosis Factor Alpha (TNF-α) in Autoimmune Disease and Current TNF-α Inhibitors in Therapeutics. Int. J. Mol. Sci..

[116] Zhu H., Liu X., Ding Y., Tan K., Ni W., Ouyang W., Tang J., Ding X., Zhao J., Hao Y. (2022). IL-6 coaxes cellular dedifferentiation as a pro-regenerative intermediate that contributes to pericardial ADSC-induced cardiac repair. Stem Cell Res. Ther..

[117] Aslan C., Kiaie S.H., Zolbanin N.M., Lotfinejad P., Ramezani R., Kashanchi F., Jafari R. (2021). Exosomes for mRNA delivery: A novel biotherapeutic strategy with hurdles and hope. BMC Biotechnol..

[118] Gorshkov A., Purvinsh L., Brodskaia A., Vasin A. (2022). Exosomes as Natural Nanocarriers for RNA-Based Therapy and Prophylaxis. Nanomaterials.

[119] Nuzzi R., Trossarello M., Bartoncini S., Marolo P., Franco P., Mantovani C., Ricardi U. (2020). Ocular Complications after Radiation Therapy: An Observational Study. Clin. Ophthalmol..

[120] Fish D.E., Kim A., Ornelas C., Song S., Pangarkar S. (2011). The risk of radiation exposure to the eyes of the interventional pain physician. Radiol. Res. Pract..

[121] Rose A., Rae W.I.D., Sweetlove M.A., Ngetu L., Benadjaoud M.A., Marais W. (2022). Radiation induced cataracts in interventionalists occupationally exposed to ionising radiation. SA J. Radiol..

